# Statins mediate anti- and pro-tumourigenic functions by remodelling the tumour microenvironment

**DOI:** 10.1242/dmm.049148

**Published:** 2022-01-04

**Authors:** Tamihiro Kamata, Esraa Al Dujaily, Salwa Alhamad, Tsz Y. So, Olga Margaritaki, Susan Giblett, J. Howard Pringle, John Le Quesne, Catrin Pritchard

**Affiliations:** 1 Leicester Cancer Research Centre, University of Leicester, Leicester Royal Infirmary, Leicester LE2 7LX, UK; 2 Department of Molecular Cell Biology, University of Leicester, Lancaster Road, Leicester LE1 9HN, UK

**Keywords:** Lung adenocarcinoma, Statins, Mouse models, Tumour microenvironment, Drug resistance

## Abstract

Anti-cancer properties of statins are controversial and possibly context dependent. Recent pathology/epidemiology studies of human lung adenocarcinoma showed reduced pro-tumourigenic macrophages associated with a shift to lower-grade tumours amongst statin users but, paradoxically, worse survival compared with that of non-users. To investigate the mechanisms involved, we have characterised mouse lung adenoma/adenocarcinoma models treated with atorvastatin. Here, we show that atorvastatin suppresses premalignant disease by inhibiting the recruitment of pro-tumourigenic macrophages to the tumour microenvironment, manifested in part by suppression of Rac-mediated CCR1 ligand secretion. However, prolonged atorvastatin treatment leads to drug resistance and progression of lung adenomas into invasive disease. Pathological progression is not driven by acquisition of additional driver mutations or immunoediting/evasion but is associated with stromal changes including the development of desmoplastic stroma containing Gr1^+^ myeloid cells and tertiary lymphoid structures. These findings show that any chemopreventive functions of atorvastatin in lung adenocarcinoma are overridden by stromal remodelling in the long term, thus providing mechanistic insight into the poor survival of lung adenocarcinoma patients with statin use.

## Abstract (precis)

**Summary:** Characterisation of mouse lung adenoma/adenocarcinoma models treated with atorvastatin revealed that statins remodel the tumour microenvironment in lung adenocarcinoma development to create pro- and anti-tumourigenic effects dependent on disease stage.

## INTRODUCTION

Statins are widely prescribed cholesterol-lowering drugs that inhibit 3-hydroxy-3-methylglutaryl coenzyme A (HMG-CoA) reductase, the key regulator of the mevalonate pathway, to promote cholesterol biosynthesis and isoprenoid production. Apart from their cholesterol-lowering function, statins have been shown to exert *in vivo* anti-cancer activity in a wide variety of animal models, and to induce cell cycle arrest or apoptosis in several cancer cell lines *in vitro* (Clendening and Penn, 2012). Isoprenoid production through the mevalonate pathway is essential for protein prenylation of RAS/RHO family small GTPases, and C-terminal prenylation of the small GTPases is a prerequisite for their membrane anchoring and activation (Wang and Casey, 2016). Suppression of small GTPase prenylation through inhibition of the mevalonate pathway has been proposed as one of the key mechanisms underpinning the anti-tumour and immunomodulatory functions of statin (Clendening and Penn, 2012; Greenwood et al., 2006).

A number of epidemiology studies have demonstrated a relationship between statin use and reduced cancer incidence/cancer-related mortality (Blais et al., 2000; Cardwell et al., 2015; Graaf et al., 2004; Nielsen et al., 2012; Poynter et al., 2005). However, a causal relationship between statin use and cancer incidence/mortality has remained elusive. Notably, secondary analyses of randomised trials, primarily conducted for investigating the prevention effects of statins on cardiovascular disorders, have failed to prove their anti-cancer effects (Bjerre and LeLorier, 2001; Cholesterol Treatment Trialists, 2010; Dale et al., 2006; Hebert et al., 1997), although this could be due to the relatively short observational periods of the majority of trials. We recently reported that statin use is associated with a reduction in CD68^+^CD163^+^ pro-tumourigenic tumour-associated macrophage (TAM) proportions in tumour parenchymal and stromal areas of human lung adenocarcinomas (Al Dujaily et al., 2020). However, this inhibitory effect was restricted to *in situ* regions and was not detected in invasive regions (Al Dujaily et al., 2020), suggesting that TAMs within invasive regions are refractory to the effects of statins. Indeed, despite the TAM reduction within *in situ* regions and a shift towards lower-grade tumours among statin users, the prognosis of the same lung adenocarcinoma patient cohort with statin use was marginally worse than that of non-users (Al Dujaily et al., 2020). This implies that any beneficial effect of statins on localised disease is cancelled out during progression to invasive adenocarcinomas.

To investigate the causal and mechanistic relationship between statin use and lung adenocarcinoma progression, we utilised two autochthonous, genetically engineered mouse models of lung tumourigenesis: one driven by BRAF^V600E^ that develops pre-malignant adenomas (Kamata et al., 2015) and the other by KRAS^G12D^ leading to adenocarcinoma development (Sutherland et al., 2014). We show that *in vivo* statin treatment inhibits early-stage lung tumour development in these models with effective suppression of TAM accumulation, mediated by abrogation of autocrine CC chemokine secretion. However, long-term treatment facilitates progression into advanced adenocarcinoma in the KRAS^G12D^ model through extensive stromal reorganisation associated with Gr1^+^ myeloid cell accumulation and tertiary lymphoid structure development. These findings provide mechanistic insight into the clinical observation of reduced incidence of lung adenocarcinoma among statin users but paradoxically poorer prognosis of lung adenocarcinoma patients with statin use (Al Dujaily et al., 2020).

## RESULTS

### Atorvastatin inhibits BRAF^V600E^-driven lung adenoma development

**Fig. 1.**
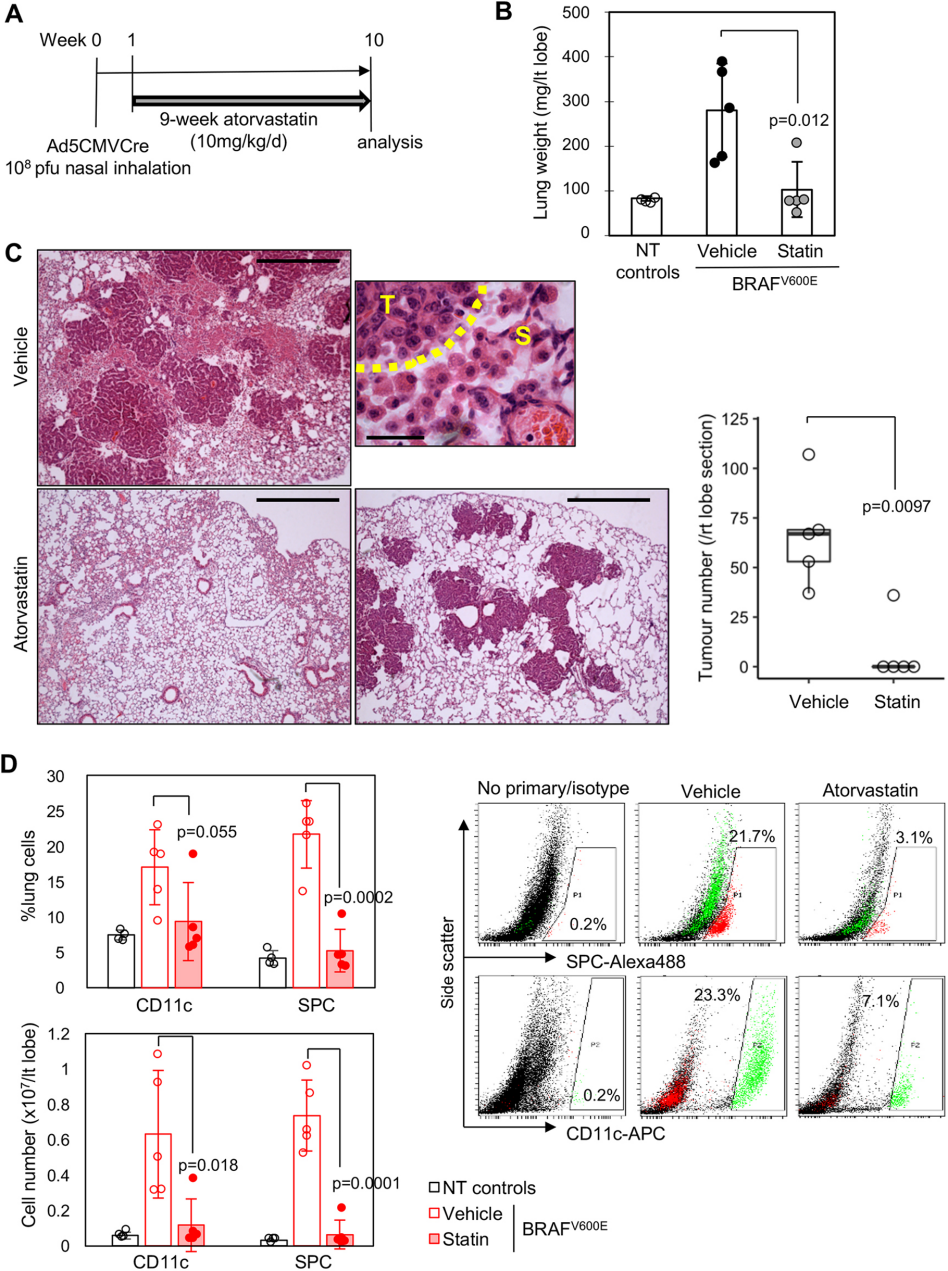
**Atorvastatin inhibits BRAF^V600E^-driven lung tumourigenesis.** (A) Schematic of atorvastatin treatment of Ad5-CMV-Cre-induced BRAF^V600E^ mice. pfu, plaque-forming units. (B) Lung weights of vehicle/atorvastatin-treated BRAF^V600E^ tumour mice. BRAF^WT^ non-tumour mice (NT) served as negative controls [*n*=4 for NT controls, *n*=5 for BRAF^V600E^ tumour mice in each treatment group, *P*-value by unpaired Student's *t*-test (two-tailed)]. (C) H&E staining of vehicle (top)/atorvastatin-treated tumours (bottom). The top-right image highlights the tumour (T)–stroma (S) border of a vehicle tumour. Scale bars: 500 µm (top left and bottom row), 25 µm (top right). The boxplot on the right shows tumour numbers per right lobe for each treatment group (*n*=5, *P*-value by Wilcoxon rank-sum test). (D) Flow cytometry analysis of CD11c^+^ tumour-associated macrophages (TAMs)/resident alveolar macrophages and SPC^+^ tumour cells/resident AT-2 cells in vehicle/atorvastatin-treated BRAF^V600E^ and BRAF^WT^ non-tumour (NT) lungs (*n*=4-5). Cell percentages (top left) and cell numbers per left lobe (bottom left) are indicated. Representative flow cytometry plots are shown on the right [*n*=4 for NT controls, *n*=5 for BRAF^V600E^ tumour mice in each treatment group, *P*-values by unpaired Student's *t*-test (two-tailed)]. Data in B and D represent mean±s.d.

We first utilised the BRAF^V600E^ mouse lung model in which premalignant adenomas develop surrounded by BRAF^WT^ stromal macrophage-lineage cells following Cre induction (Kamata et al., 2015). Tumour development was induced by nasal delivery of Ad5-CMV-Cre, followed by 9 weeks of atorvastatin treatment (Fig. 1A). Almost complete inhibition of tumour development and normalisation of lung weights was observed in atorvastatin-treated mice, whereas vehicle-treated mice developed papillary adenomas surrounded by luminal infiltration of tumour-associated macrophage-lineage cells as previously reported (Kamata et al., 2015, 2017) (Fig. 1B,C). Interestingly, modest development of papillary adenomas was observed in one drug-treated mouse, but the tumours were rarely associated with stromal macrophage-lineage cells (Fig. 1C). Quantitative analysis of CD11c^+^ (also known as ITGAX^+^) stromal immature macrophage lineage cells (IMCs) and surfactant protein-C (SPC^+^; also known as SFTPC^+^) adenoma cells using flow cytometry (Kamata et al., 2015, 2020) confirmed that both cell types were robustly decreased by atorvastatin treatment (Fig. 1D).

**Fig. 2.**
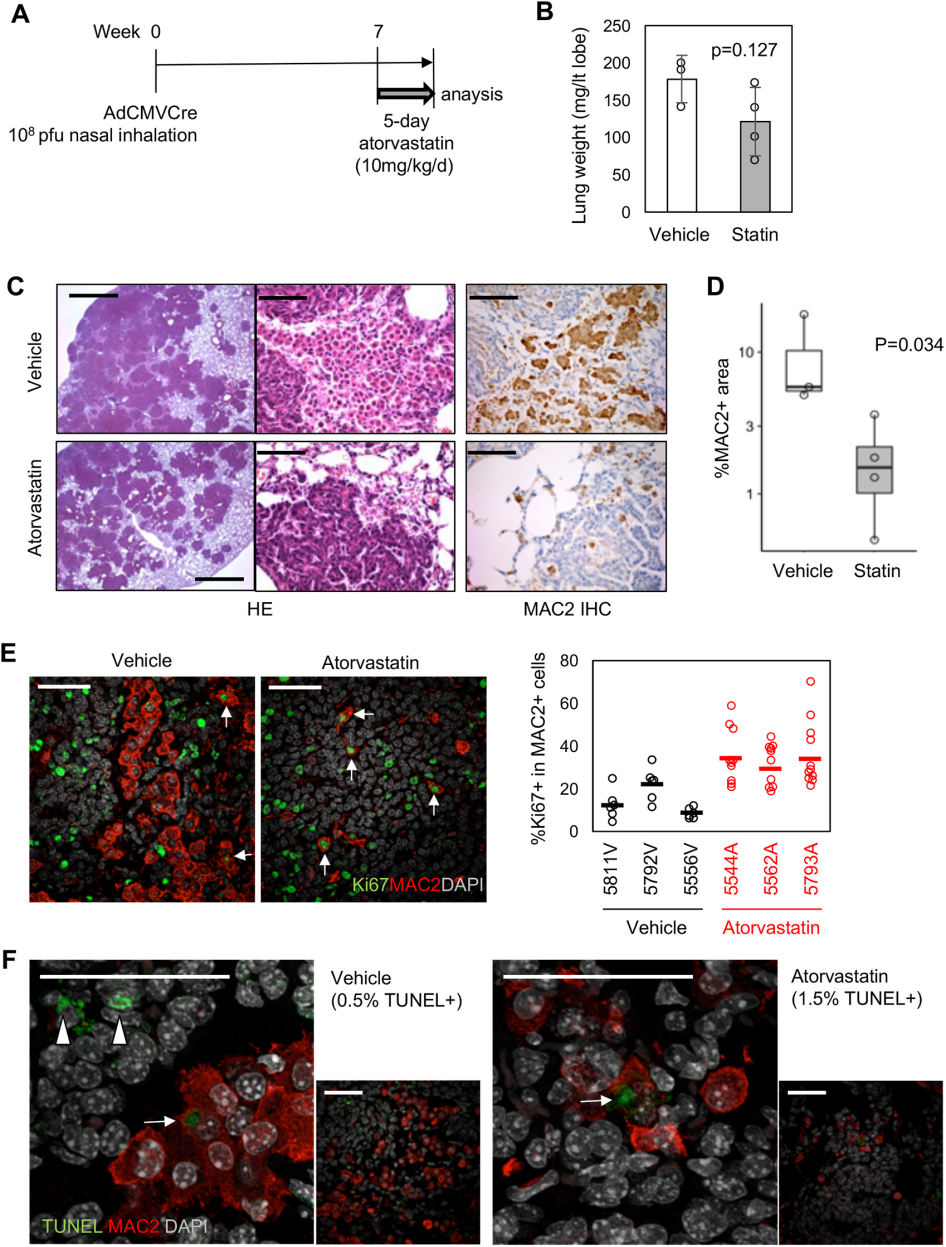
**Atorvastatin targets TAMs in BRAF^V600E^-driven tumours.** (A) Schematic of short-term atorvastatin treatment of Ad5-CMV-Cre-induced BRAF^V600E^ mice. (B) Lung weight of vehicle/atorvastatin-treated BRAF^V600E^ tumour mice [*n*=3 for vehicle controls, *n*=4 for statin treatment, *P*-value by unpaired Student's *t*-test (two-tailed)]. Data represent mean±s.d. (C) Histological analysis. Low (left)- and high (middle)-magnification H&E, and MAC2-IHC (right). Scale bars: 1 mm (left column), 62.5 µm (middle and right columns). (D) %MAC2-stained area in total lung area presented by the boxplot for each treatment group [*n*=3 for vehicle controls, *n*=4 for statin treatment, *P*-value by unpaired Student's *t*-test (two-tailed)]. (E,F) Confocal imaging of MAC2^+^ cells co-stained for Ki67 (E) or TUNEL (F). Arrows indicate Ki67^+^MAC2^+^ cells (E) or TUNEL^+^MAC2^+^ cells (F). Arrowheads indicate TUNEL^+^MAC2^−^ apoptotic tumour cells. Scale bars: 50 µm. The graph in E shows %Ki67^+^ in MAC2^+^ cells in 6-11 high-power fields per sample, individually plotted with mean values (bars). %TUNEL^+^ cells in total MAC2^+^ cells are indicated in F.

To gain further insight into the cell population(s) targeted by atorvastatin, we next treated BRAF^V600E^-expressing mice with atorvastatin over a shorter time frame (Fig. 2A). Under these conditions, atorvastatin treatment did not reduce lung weights or tumour burden (Fig. 2B,C). However, MAC2^+^ (also known as LGALS3^+^) IMCs (Kamata et al., 2015) were significantly reduced (Fig. 2C,D), suggesting that these cells are the primary target of atorvastatin *in vivo*. The residual MAC2^+^ cells in the atorvastatin-treated mice contained more Ki67^+^ (also known as Mki67^+^) cells than control mice (Fig. 2E), indicating that the reduction in stromal IMCs is unlikely to be caused by inhibition of their local proliferation. MAC2^+^ cells in this model rarely showed apoptotic nuclear morphologies (<2%), regardless of treatment, which was further confirmed by terminal deoxynucleotidyl transferase dUTP nick end labelling (TUNEL) staining (Fig. 2F). These data argue that atorvastatin inhibits recruitment and/or retention of non-proliferative MAC2^+^ cells without affecting their apoptosis, resulting in the relative enrichment of MAC2^+^ resident (alveolar) macrophages with proliferative capability. In line with these *in vivo* observations, purified IMCs were non-proliferative in culture, regardless of atorvastatin treatment (Fig. S1A,B). Interestingly, IMCs treated with atorvastatin *ex vivo* underwent cell death without showing early apoptotic changes (Fig. S1C), suggesting that atorvastatin directly causes non-apoptotic death of IMCs.

Other inflammatory immune cells are detected in this model, particularly T and B lymphocytes (Kamata et al., 2015), which occasionally form tumour-associated tertiary lymphoid structures (TA-TLS) (Sautès-Fridman et al., 2019) (Fig. S2A). TA-TLS development was not inhibited by short-term atorvastatin treatment (Fig. S2A). However, interestingly, TA-TLS in control mice were mostly B-cell rich, whereas more T cells were involved in the TA-TLS in atorvastatin-treated mice (Fig. S2B). Nevertheless, intratumour T-cell infiltration was rarely observed in either control or atorvastatin-treated mice (Fig. S2C); therefore, it is unlikely that atorvastatin exerts its anti-tumour functions through modulating T-cell immunity.

### Atorvastatin suppresses TAM accumulation in the KRAS^G12D^-driven model

**Fig. 3.**
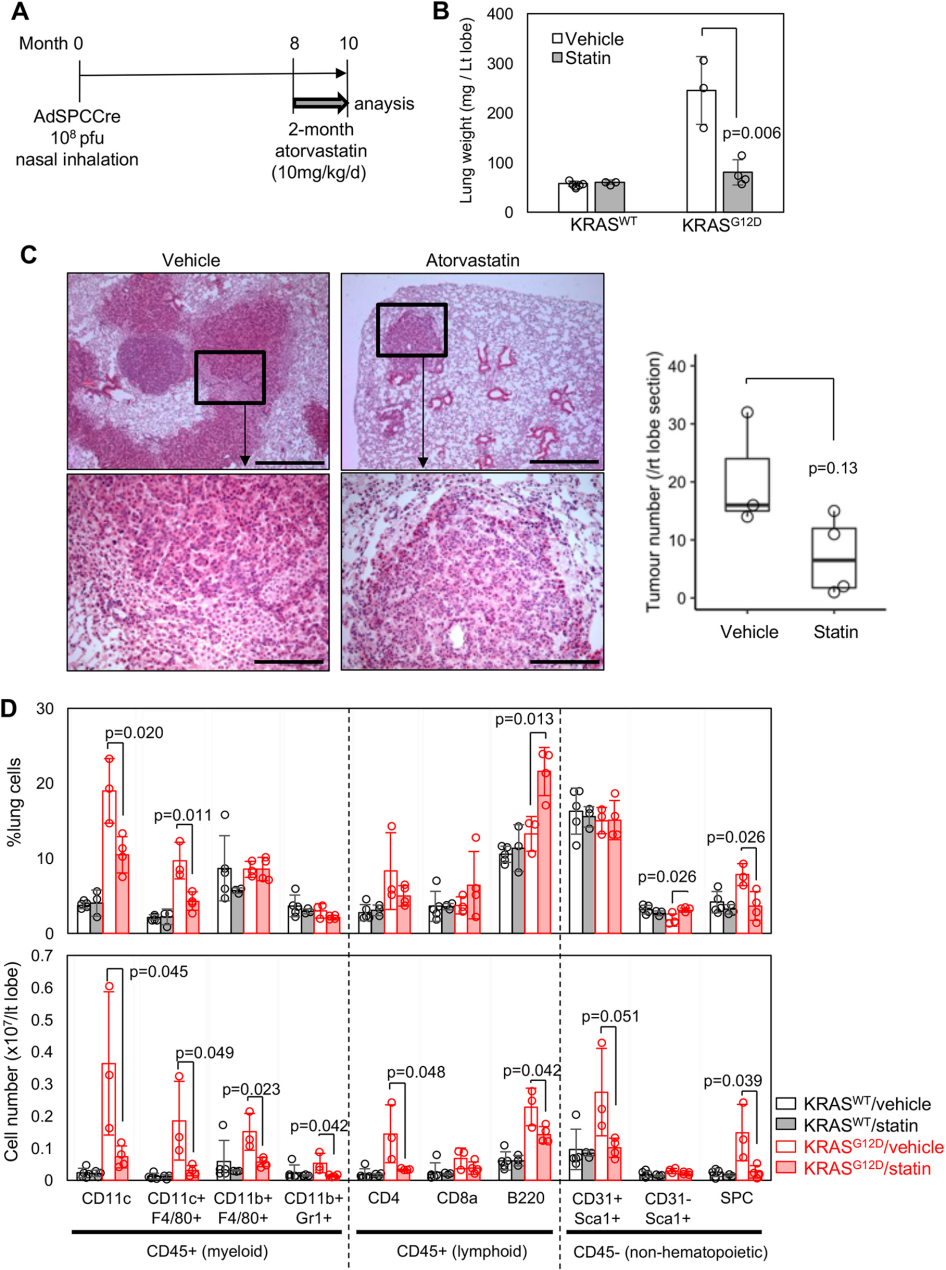
**Short-term atorvastatin treatment.** (A) Schematic of 2-month atorvastatin treatment of Ad5-mSPC-Cre-induced KRAS^G12D^ mice. (B) Lung weight of vehicle/atorvastatin-treated KRAS^G12D^ tumour mice [*n*=5 for KRAS^WT^ vehicle groups, *n*=3 for KRAS^WT^ statin and KRAS^G12D^ vehicle groups, *n*=4 for KRAS^G12D^ statin group, *P*-value by unpaired Student's *t*-test (two-tailed)]. KRAS^WT^ mice serve as negative controls. (C) H&E staining. The boxed areas in the top images are enlarged in the bottom images. Scale bars: 500 µm (top row), 125 µm (bottom row). The boxplot on the right shows tumour numbers per right lobe for each treatment group [*n*=3 for vehicle control, *n*=4 for statin treatment, *P*-value by unpaired Student's *t*-test (two-tailed)]. (D) Flow cytometry analysis of myeloid (CD11c^+^, CD11c^+^F4/80^+^, CD11b^+^F4/80^+^, CD11b^+^Gr1^+^), lymphoid (CD4^+^, CD8a^+^, B220^+^) and non-hematopoietic (CD31^+^Sca1^+^, CD31-Sca1^+^, SPC^+^) populations in vehicle/atorvastatin-treated KRAS^WT^/KRAS^G12D^ lungs (*n*=3-4). Cell percentages (top) and cell numbers per left lobe (bottom) are indicated [*n*=5 for KRAS^WT^ vehicle groups, *n*=3 for KRAS^WT^ statin and KRAS^G12D^ vehicle groups, *n*=4 for KRAS^G12D^ statin group, *P*-values by unpaired Student's *t*-test (two-tailed)]. Data in B and D represent mean±s.d.

We infected *Kras^+/LSL−G12D^* mice with the Ad5-mSPC-Cre adenoviral vector (Kamata et al., 2020; Sutherland et al., 2014) and allowed premalignant adenomas to develop for 8 months (Fig. 3A) (Kamata et al., 2020). Atorvastatin/vehicle treatments were then undertaken for 8 weeks. Lung weights were significantly reduced compared with control levels (Fig. 3B), and histological analysis showed reduction of tumour burden in atorvastatin-treated mice (Fig. 3C), although the histopathological characteristics of the adenomas were not affected (Fig. S3A). Of note, atorvastatin marginally reduced tumour number in this model, but did not completely abrogate the tumours (Fig. 3C), in stark contrast to the BRAF model (Fig. 1C), suggesting that the inhibitory effects of atorvastatin could in part depend on the timing of the treatment. Flow cytometry quantitation showed significant decreases in the number and percentage of SPC^+^ tumour cells by atorvastatin (Fig. 3D; Fig. S3B). In addition, peri-tumour stroma development was strongly suppressed (Fig. 3C; Fig. S3A), and most of the stromal cell types examined by flow cytometry were significantly decreased by atorvastatin (Fig. 3D). Of note, CD11c^+^ macrophage-lineage cells, including F4/80^−^ (also known as ADGRE1^+^) cells that are equivalent to IMCs in the BRAF^V600E^ model (Kamata et al., 2015), and TAM-like F4/80^+^ cells (Kamata et al., 2020; Franklin et al., 2014) were the sole cell populations in the stroma significantly decreased by atorvastatin when compared as relative percentages (Fig. 3D), suggesting that, as with the BRAF^V600E^ model, these are the primary cell targets of atorvastatin.

### Atorvastatin disrupts autocrine CCR1 signalling

**Fig. 4.**
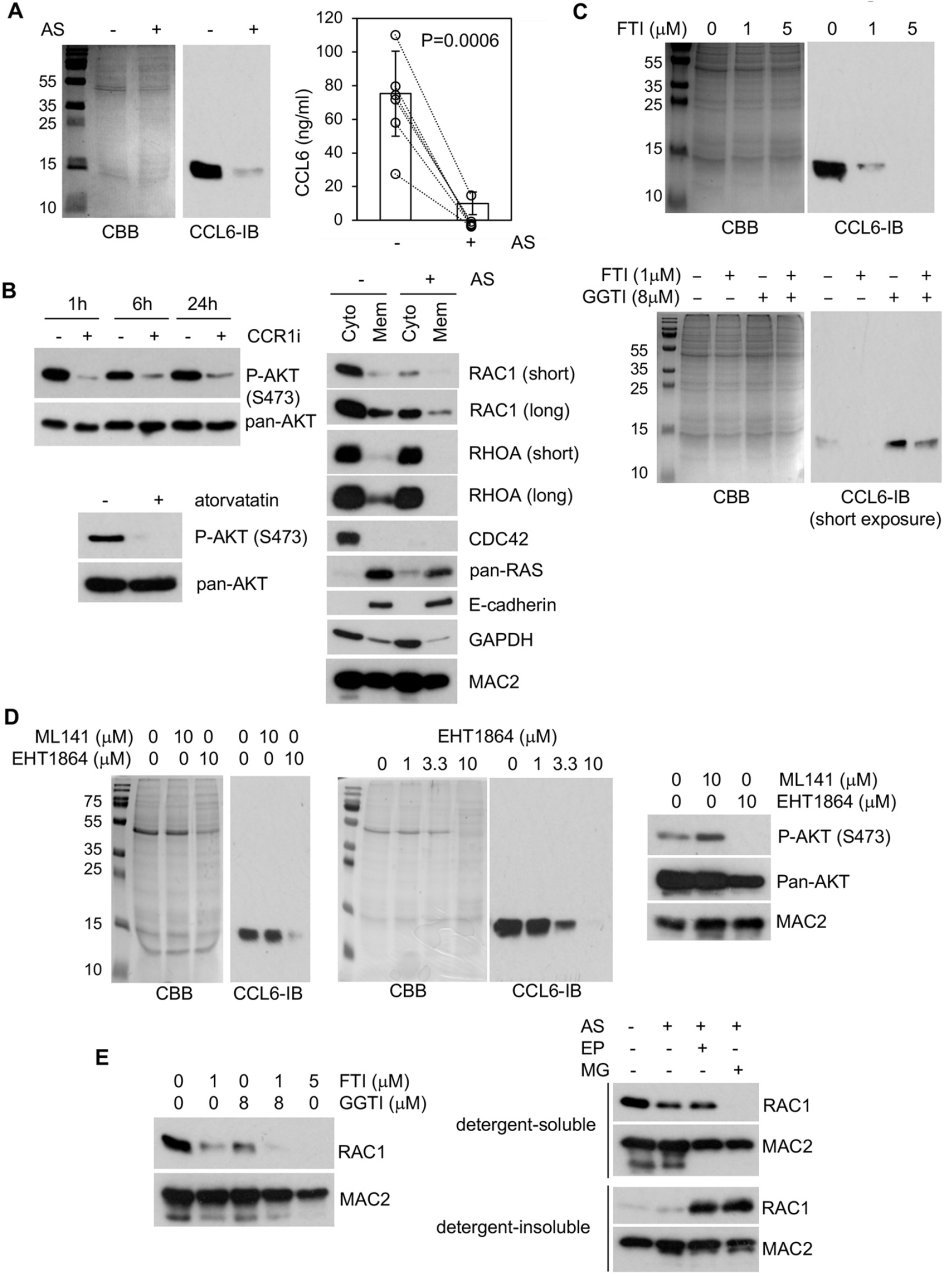
**Atorvastatin inhibits CCL6 secretion.** (A) Immunoblotting (left) and ELISA (right) of CCL6 in CM of 72 h immature macrophage lineage cell (IMC) culture±3.3 µM atorvastatin (AS). The immunoblot represents six biological replicates showing similar results. For the ELISA, IMC cultures were established from six independent tumour mice and data represent mean±s.d. (B) Phospho-AKT immunoblotting of IMC lysates obtained after 96 h culture followed by 1-24 h CCR1 inhibitor (CCR1i; 5 µM J113863) treatment (top left) or 72 h culture±3.3 µM AS (bottom left). Pan-AKT blots served as loading controls. The right panels show immunoblotting of RHOA/RAC1/CDC42/pan-RAS in cytosolic (Cyto)/membrane (Mem) fractions of lysates from IMCs cultured for 48 h±3.3 µM AS+1% foetal bovine serum. E-cadherin, GAPDH and MAC2 served as membrane, cytoplasmic and ubiquitous protein loading controls, respectively. The immunoblots represent three biological replicates showing similar results. (C) Immunoblotting of CCL6 in conditioned medium (CM) of 72 h IMC culture with farnesyltransferase inhibitor (FTI; lonafarnib) (top) or FTI/GGTI-298 combination (bottom). The immunoblots represent three biological replicates showing similar results. (D) Immunoblotting of CCL6 in CM of 72 h IMC culture with ML141/EHT1864 (left) or EHT1864 (middle). The right panel shows immunoblotting of IMC lysates after 72 h culture with ML141/EHT1864. Pan-AKT/MAC2 blots served as loading control. The immunoblots represent three biological replicates showing similar results. (E) RAC1 immunoblotting of IMCs lysates after 72 h culture with FTI/GGTI-298 (left) or 72 h culture with proteasome inhibitors Epoxomicin (EP) or MG132 (MG)+AS (right). MAC2 blots served as loading controls. The immunoblots represent three biological replicates showing similar results. Coomassie Brilliant Blue (CBB) staining served as loading control for panels A, C and D.

The above data suggest that suppression of tumour-associated macrophage lineage cells by atorvastatin is not mediated through proliferative/apoptotic mechanisms; therefore, we investigated whether the drug acts on pathways involved in recruitment of these cells to the tumour microenvironment (TME). We have previously shown that autocrine CC chemokine receptor 1 (CCR1) signalling plays a critical role in IMC accumulation in the BRAF^V600E^-driven lung adenoma model (Kamata et al., 2015). To investigate this further, IMCs purified from *Braf^+/LSL−V600E^;CreER^+/0^* mice were treated with atorvastatin *ex vivo*. Secretion of CC chemokine 6 (CCL6), an autocrine CCR1 ligand produced by IMCs (Kamata et al., 2015), was drastically decreased by atorvastatin (Fig. 4A). AKT phosphorylation is largely dependent on autocrine CCR1 signalling in IMCs and was also effectively inhibited by atorvastatin (Fig. 4B).

Statins suppress protein prenylation (Wang and Casey, 2016); therefore, we investigated the link between CCL6 secretion and the two forms of protein prenylation, farnesylation and geranylgeranylation. CCL6 secretion was inhibited by the farnesyltransferase inhibitor (FTI) lonafarnib but the geranylgeranyltransferase-I (GGTase-I) inhibitor GGTI-298 enhanced CCL6 secretion (Fig. 4C). Further analysis showed that expression of cytoplasmic and membrane-localised RHO-family small GTPases RAC1 and CDC42 were robustly reduced by atorvastatin, whereas RhoA and pan-RAS proteins were not affected (Fig. 4B). Thus, we tested the effects of the RAC1/CDC42 inhibitors directly. The RAC inhibitor EHT1864 reduced CCL6 secretion in a dose-dependent manner and inhibited autocrine AKT activation, whereas CDC42 inhibition by ML141 suppressed neither (Fig. 4D). RAC1 expression was reduced by lonafarnib and, to a lesser extent, by GGTI-298 (Fig. 4E). Furthermore, RAC1 accumulation was detected following proteasome inhibition in the detergent-insoluble protein fraction of atorvastatin-treated IMCs (Fig. 4E). Collectively, our data suggest that inhibition of protein prenylation and proteasomal degradation of RAC1 in IMCs contributes, at least in part, to the suppression of CCL6 secretion by atorvastatin.

### Long-term atorvastatin treatment induces progression to invasive adenocarcinoma

**Fig. 5.**
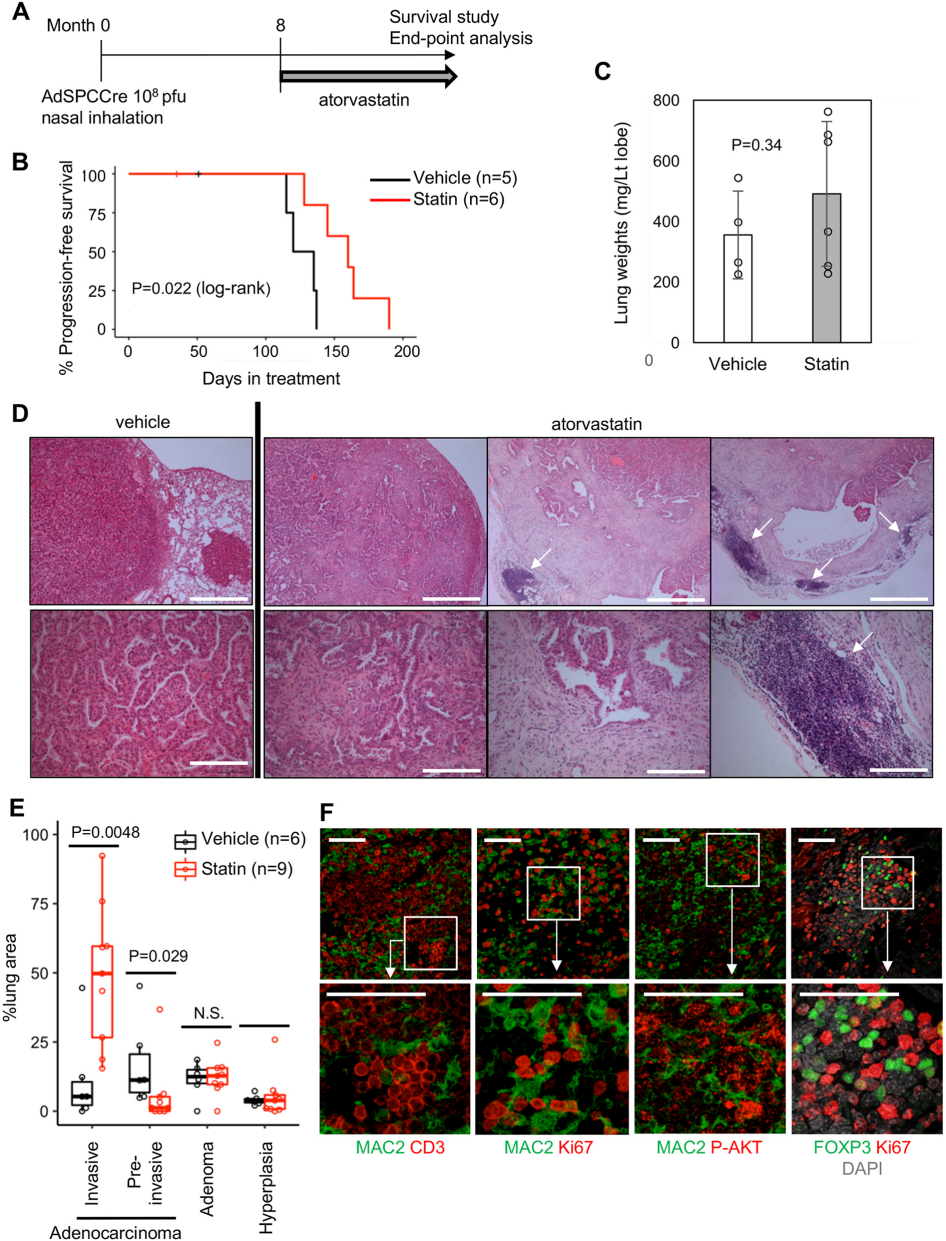
**Long-term atorvastatin treatment facilitates malignant progression.** (A) Schematic of long-term atorvastatin treatment of Ad5-mSPC-Cre-induced KRAS^G12D^ mice. (B) Survival of KRAS^G12D^ mice following vehicle (*n*=5)/atorvastatin (*n*=6) treatment (*P*-value by χ^2^ test). (C) Lung weights at endpoint of vehicle (*n*=4)/atorvastatin-treated (*n*=6) KRAS^G12D^ mice [*P*-value by unpaired Student's *t*-test (two-tailed)]. Data represent mean±s.d. (D) H&E staining of vehicle- or atorvastatin-treated tumours at endpoint. Scale bars: 500 µm (top row), 125 µm (bottom row). Arrows indicate tumour-associated tertiary lymphoid structures (TA-TLS). (E) %tumour areas with different histological grades (hyperplasia, adenoma, invasive and pre-invasive adenocarcinoma) were quantitated and presented by the boxplot for each treatment group (*n*=6 for vehicle controls, *n*=9 for statin treatment, *P*-values by Wilcoxon rank-sum test). N.S., not significant. (F) Confocal imaging of TA-TLS in atorvastatin-treated tumours for MAC2/FOXP3 (green) and CD3/Ki67/phospho-AKT (P-AKT) (red). The boxed areas in the top images are enlarged in the bottom images. The images represent three biological replicates showing similar results. Scale bars: 50 µm.

KRAS^G12D^-driven adenomas progress into adenocarcinomas at later time points (Sutherland et al., 2014); therefore, we extended the treatment period to investigate long-term atorvastatin treatment (Fig. 5A). Although their survival time was significantly extended when compared with that of vehicle-treated mice (Fig. 5B), all atorvastatin-treated mice showed fatal respiratory failure within 200 days. Lung weights were comparable to those of vehicle-treated mice (Fig. 5C), and tumour development with advanced histology was confirmed in all atorvastatin-treated mice (Fig. 5D,E). Tumours in vehicle-treated mice predominantly showed non-invasive (*in situ*) characteristics, whereas tumours in atorvastatin-treated mice exhibited invasive features with higher Ki67 positivity (Fig. 5E; Fig. S4) and were often accompanied by extensive development of desmoplastic stroma and TA-TLS (Fig. 5F). The burden of pre-malignant lesions (adenoma/hyperplasia) was not affected by the long-term atorvastatin treatment (Fig. 5E). However, there was an increased burden of invasive disease (Fig. 5E), suggesting that long-term atorvastatin treatment induces histological progression of adenocarcinoma. Interestingly, AKT phosphorylation was induced at the tumour–stroma interface of invasive atorvastatin-treated tumours (Fig. S5), suggesting that tumour–stroma interactions contribute to re-activation of RAS downstream pathways as previously reported (Kruspig et al., 2018), leading to histopathological progression.

TA-TLS in this model were identified as T cell-rich lymphocytic clusters (Fig. 5F), in a similar manner to those in the BRAF^V600E^ model (Fig. S1) and were associated with MAC2^+^ interdigitating dendritic cells (DCs) (Flotte et al., 1983). Some cells directly contacting the MAC2^+^ DCs were positive for Ki67 and phospho-AKT (P-AKT) (Fig. 5F), suggesting that T cells in the TA-TLS are activated by interdigitating DCs as reported (Goc et al., 2014) and that atorvastatin did not inhibit AKT activation in these cells *in vivo*. Forkhead box P3 (FOXP3)^+^ regulatory T cells (Tregs) were enriched in the TA-TLS (Fig. 5F) as reported (Williams et al., 2016), and some FOXP3^+^ nuclei (25.9% on average) were co-stained for Ki67, showing the phenotype of tumour-associated proliferating Tregs (Plitas et al., 2016).

### Ly6C^+^ monocytic cells are expanded by long-term atorvastatin treatment

**Fig. 6.**
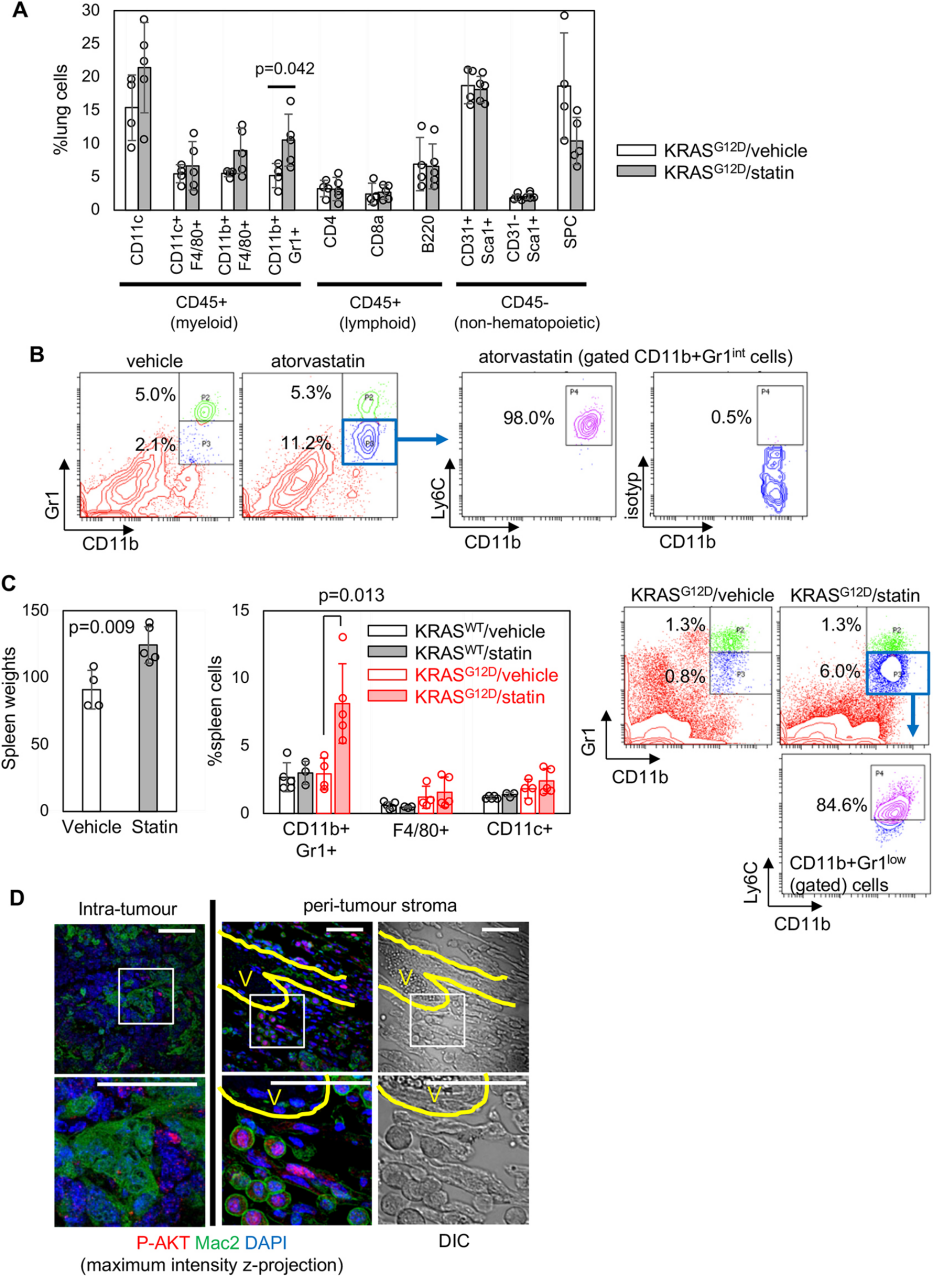
**Myeloid-derived suppressor cell accumulation in atorvastatin-treated lungs.** (A) Flow cytometry analysis at endpoint [*n*=4 for vehicle controls, *n*=5 for statin treatment, *P*-value by unpaired Student's *t*-test (two-tailed)]. (B) Representative flow cytometry plots for CD11b^+^Gr1^int^Ly6C^+^ cells in atorvastatin-treated KRAS^G12D^ lungs, including isotype staining to determine the background fluorescence of CD11b^+^Gr1^int^ cells. (C) Endpoint spleen weights of vehicle/atorvastatin-treated KRAS^G12D^ mice (left) and flow cytometry quantitation of splenic CD11bGr1^+^, F4/80^+^ and CD11c^+^ cells at endpoint (middle) [*n*=5 for KRAS^WT^/vehicle and KRAS^G12D^/statin groups, *n*=3 for KRAS^WT^/statin group, *n*=4 for KRAS^G12D^/vehicle group, *P*-values by unpaired Student's *t*-test (two-tailed)]. Representative flow cytometry plots for CD11b/Gr1/Ly6C expression are shown on the right. (D) Confocal imaging of the intratumour/peri-tumour MAC2^+^ cells co-stained for P-AKT. Maximum intensity *z*-projection images are shown with differential interference contrast (DIC) images of cellular morphologies in the peri-tumour stroma. The endothelial lining of microvessels (V) is highlighted in yellow. The boxed areas in the top images are enlarged in the bottom images. Scale bars: 50 µm. The images represent three biological replicates showing similar results. Data in A, C and D represent mean±s.d.

To further investigate the paradoxical stromal response to atorvastatin, we performed flow cytometry quantitation at endpoint. This analysis demonstrated a significant increase in the relative percentage of CD11b^+^ (also known as ITGAM^+^)Gr1^+^ myeloid cells in atorvastatin-treated KRAS^G12D^ lungs, whereas there were no significant differences in other populations (Fig. 6A). In particular, the CD11b^+^Gr1^int^Ly6C^+^ population was drastically expanded (Fig. 6B), suggesting that Ly6C^+^ monocytes/monocytic (M)-myeloid-derived suppressor cells (MDSCs) (Olingy et al., 2019) play a role in atorvastatin-mediated stromal remodelling. In line with the fact that systemic MDSC accumulation is often observed in peripheral lymphoid organs (Kumar et al., 2016), atorvastatin-treated KRAS^G12D^ mice showed splenomegaly with increased CD11b^+^Gr1^int^Ly6C^+^ cells in the spleen (Fig. 6C).

Because atorvastatin inhibits autocrine AKT phosphorylation in IMCs *ex vivo* (Fig. 4), we investigated whether long-term atorvastatin treatment affects AKT phosphorylation in tumour-associated myeloid populations *in vivo*. To this end, KRAS^G12D^ tumours treated with atorvastatin were immunostained for P-AKT and MAC2, the latter of which was used as a marker for both CD11c^+^ IMC/TAMs (Kamata et al., 2015, 2020) and Ly6C^+^ monocytes/M-MDSCs (Movahedi et al., 2008; Yu et al., 2016). Intratumour MAC2^+^ cells showed amoeboid macrophage morphologies, whereas MAC2^+^ cells located near blood vessels in the peri-tumour stroma showed a monocyte-like round morphology (Fig. 6D). In contrast to intratumour MAC2^+^ cells that were negative for P-AKT, peri-tumour MAC2^+^ round cells were mostly P-AKT^+^ (Fig. 6D), suggesting that atorvastatin inhibits P-AKT in intratumour TAMs, but not in peri-tumour monocytes/M-MDSCs accumulated by extravasation through tumour microvessels.

### Atorvastatin-induced tumour progression is not associated with additional driver mutations

We also performed whole-exome sequencing (WES) to investigate whether additional mutations accompany acquired atorvastatin resistance. Tumours in control mice were variable in size, whereas the KRAS^G12D^ mice under statin treatment developed single large tumours (Fig. 5D; Fig. S6). Accordingly, we selected moderate-sized (5498V) and large-sized (5493V) tumours for control samples, and three large tumours (5509A, 5492A, 5522A) for statin-treated samples.

**Fig. 7.**
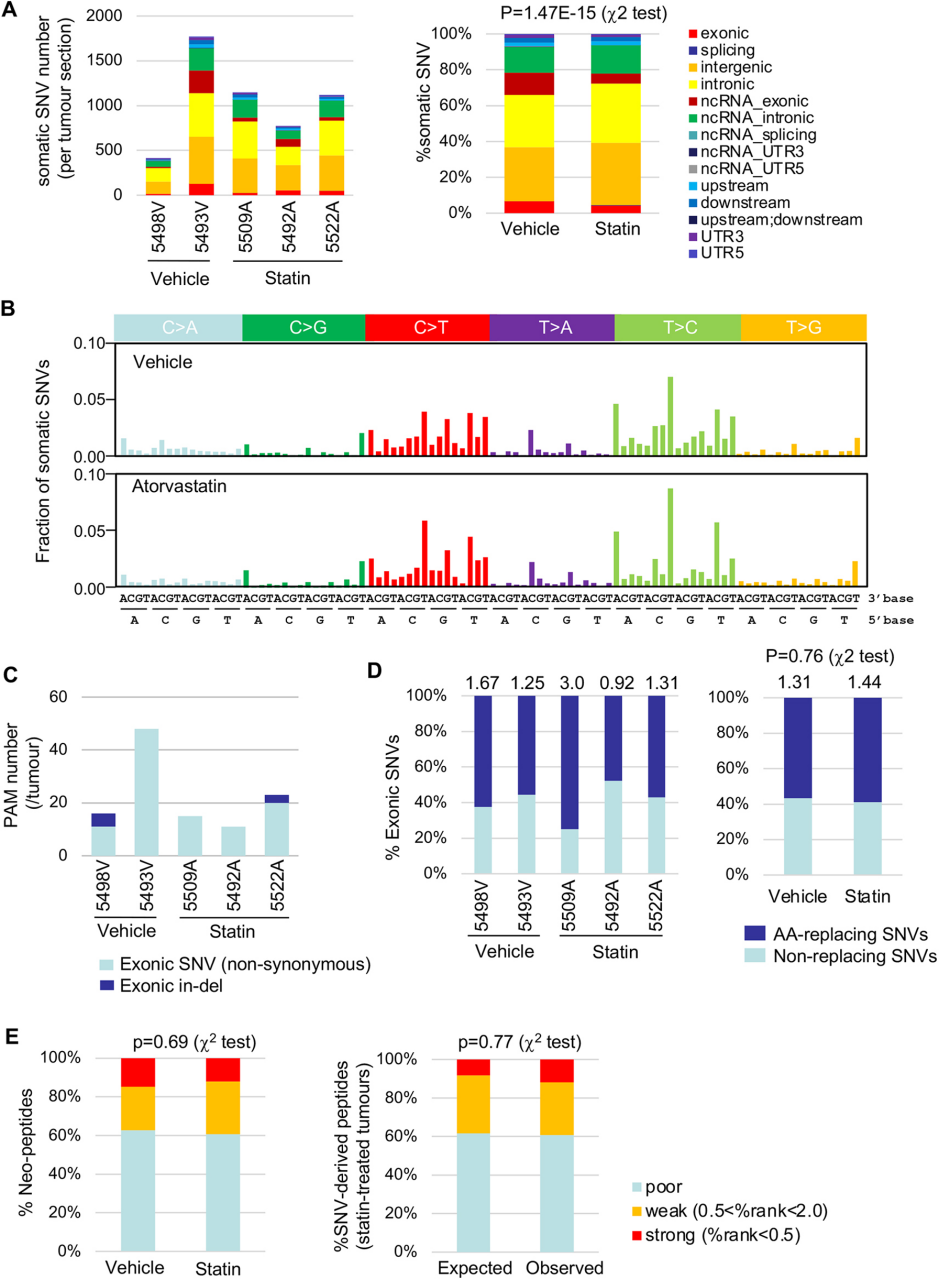
**Whole-exome sequencing of KRAS^G12D^-driven lung adenocarcinomas.** (A) Somatic single-nucleotide variant (SNV) distributions in distinct genomic regions, expressed as absolute number of SNVs for each tumour (left) or as relative percentage in total SNVs in vehicle/statin groups (right). Upstream/downstream are defined as the regions within 1 kb from the transcription start/termination sites. (B) 96-trinucleotide mutational spectra of total somatic SNVs in vehicle (top)/atorvastatin (bottom)-treated tumours. (C) Protein-altering mutation (PAM) number per tumour. (D) Distribution (%) of amino acid (AA)-replacing and non-replacing mutations in exonic somatic SNVs in each tumour (left) and in vehicle/statin groups (right). The ratios of AA-replacing to non-replacing mutations are indicated at the top of the graphs. (E) MHC class-I (H-2Kb/H-2Db) binding prediction (left) of SNV-derived neo-peptides in vehicle/atorvastatin-treated tumours, categorised into poor/weak/strong binders according to the %rank thresholds defined by NetMHCpan4.1, and comparison of predicted MHC binding of neo-peptides (right) in atorvastatin-treated tumours to that expected from silent (synonymous) SNVs in the same tumours. *P*-values for A, D and E are calculated by χ^2^ test.

We obtained an average of 94.8 million reads per sample with a median average depth of 107x (range 82-152x), and 70% of the reads (range 66-73%) were on or near (within 200 bp upstream/downstream) the target regions. Our analysis including the reads outside the targets (Guo et al., 2012) resulted in identification of single-nucleotide variants (SNVs)/InDels in non-coding regions together with exonic alterations (Fig. 7A; Table S1). In vehicle tumours, more somatic SNVs were found in the larger tumour (5493V), whereas the statin tumours consistently showed fewer somatic SNVs (Fig. 7A). Notably, statin treatment significantly reduced the fraction of exonic SNVs without robustly affecting their base substitution spectra (Fig. 7A,B), although the mutational signature contribution was variable among the tumours (Fig. 5C). Within these exonic SNVs/InDels, 11-48 protein-altering mutations (PAMs), including non-synonymous SNVs and InDels in coding regions, were found in each tumour (Fig. 7C; Table S1). None of the PAMs have previously been detected in similar KRAS^G12D^-driven mouse lung adenocarcinoma models (McFadden et al., 2016), and *Clcc1* was the only gene recurrently mutated in our cohort (p.A401V in 5498V, p.D449Efs*22 in 5522A, Table S1). However, as *CLCC1* mutations are rare in human lung adenocarcinoma (0.35%, TCGA PanCancer Atlas; https://www.cancer.gov/about-nci/organization/ccg/research/structural-genomics/tcga) and they have an even distribution pattern (COSMIC; https://cancer.sanger.ac.uk/cosmic), we did not consider *Clcc1* mutations as secondary drivers.

### No evidence of immunoediting in atorvastatin-resistant tumours

Tumour neo-antigens derived from somatic SNVs can be recognised by adaptive immunity, leading to tumour cell elimination by immune cytolytic activity (Rooney et al., 2015) or immune evasion through neo-antigen depletion at the genomic and/or transcriptional levels (Rosenthal et al., 2019). Such immune pressure may have caused the reduction of exonic SNVs under atorvastatin treatment (Fig. 7A). To examine this possibility, we first compared the frequency of amino acid-replacing SNVs with silent exonic mutations (synonymous or stop mutations). Because the latter mutations are expected to be stable against immune pressure due to the low avidity of self-reactive T cells (Pedersen et al., 2013), they served as an internal control to evaluate the impact of immune pressure on non-synonymous SNVs. The ratio of non-synonymous to synonymous/stop-gain SNVs in statin-treated tumours was similar to that in controls (Fig. 7D), suggesting no evidence for altered immune pressure by atorvastatin.

We next utilised NetMHCpan-4.1 (Reynisson et al., 2020) to predict major histocompatibility complex (MHC) class I binding to the neo-peptides derived from the non-synonymous SNVs. On average, ∼40% of the neo-peptides were predicted to function as neo-antigens (%rank<2.0), but no significant difference in the distribution of the %rank scores was observed between control and statin tumours (Fig. 7E; Fig. S7A and Table S1). The fraction of strong MHC binders (%rank<0.5) was modestly reduced in statin tumours but was larger than the theoretical expected rate using silent mutations (Rooney et al., 2015) (Fig. 7E; Tables S2 and S3). Furthermore, no correlation was observed between the variant allele frequencies (VAFs) and %rank scores of the SNVs in the statin tumours (Fig. S7B), suggesting a lack of immune pressure to reduce SNV-derived neo-antigens. In addition, the linear relationship between the cumulative SNV number and the inverse VAF in statin tumours (Fig. S7C,D) fitted the neutral evolution model (Williams et al., 2016), indicating that these tumours were not associated with selective clonal evolution linked to immune evasion. These data demonstrate that the tumours developed under long-term atorvastatin treatment are naïve to immune selection pressure and that T cells accumulated in TA-TLS in the statin tumours (Fig. 5F) do not efficiently contribute to immune surveillance.

## DISCUSSION

We recently demonstrated that statin use is associated with reduced TAMs within *in situ* lesions of human lung adenocarcinoma and reduced grade, but the survival outcome for statin users amongst the same cohort was puzzlingly worse (Al Dujaily et al., 2020). In the present study, we have tackled the underlying causes of this paradoxical observation using autochthonous mouse models. Our data demonstrate that atorvastatin has inhibitory effects on TAM populations rather than tumour cells and that the inhibitory effects are restricted to early-stage adenoma models. Furthermore, we provide evidence of acquired resistance to atorvastatin that is not associated with tumour intrinsic changes in mutational burden but is driven by stromal remodelling, leading to progression of KRAS^G12D^-driven lung adenomas into invasive adenocarcinomas. These observations raise cautionary notes regarding the clinical use of atorvastatin.

The reasons for the exquisite susceptibility of TAMs to atorvastatin in early lung disease (Figs 1–3) are not entirely clear; extensive data using human tumour cell lines with oncogenic mutations suggest that statins have direct anti-tumour functions. However, the physiological dose of statins may be relevant here because the cell line experiments frequently rely on excess doses of statins, whereas we attempted to use doses equivalent to those used clinically. Atorvastatin administered to patients ranges from 0.167 mg/kg/day to 1.33 mg/kg/day, achieving plasma concentrations of 0.05-0.12 µM (Corsini et al., 1999). Administration of 10 mg/kg atorvastatin oral doses to rats [equivalent to 19.8 mg/kg for mice, according to body surface area-based adjustments (Nair and Jacob, 2016)] has been reported to achieve 0.21 µM plasma concentration (Lau et al., 2006), hence our reasoning that 10 mg/kg/day oral dosing to mice likely achieves plasma concentrations within or very close to the range reported for patients.

We previously reported that IMCs in the BRAF^V600E^ model are phenotypically similar to monocyte-derived alveolar macrophages (Mo-AMs) (Kamata et al., 2020) and are regulated by autocrine CCR1 signalling (Kamata et al., 2015). Here, we find that atorvastatin suppresses IMC secretion of CCL6, thereby inhibiting autocrine CCR1 activation to disrupt IMC accumulation. Atorvastatin likely exerts this inhibitory effect by facilitating proteasome degradation of RAC1 that plays a vital role in inflammatory cytokine secretion by macrophages (Akula et al., 2019; Fu et al., 2019; Stanley et al., 2014). Interestingly, FTI recapitulated the inhibitory effects of atorvastatin, whereas GGTI rather enhanced CCL6 secretion, consistent with the hyperactive phenotype of GGTase-I-deficient or GGTI-treated macrophages (Akula et al., 2019; Fu et al., 2019). Because RAC1 is a GGTase-I substrate (Kinsella et al., 1991), our data suggest that inhibition of protein farnesylation by atorvastatin or FTI does not directly target RAC1 prenylation but may affect RAC1 protein stability by promoting defarnesylation of RAC1-interacting proteins. Further investigation of the mechanisms for RAC1 degradation by atorvastatin are now warranted.

We speculate that the acquired stromal resistance to atorvastatin stems from TAM switching from CCR1-dependent Mo-AMs/IMCs (Kamata et al., 2015, 2020) to monocytes/M-MDSC-derived TAMs recruited by CCR2 signalling (Kumar et al., 2016; Olingy et al., 2019). Indeed, Gr1^+^ MDSCs have previously been reported to play a role in mediating stromal resistance to BRAF inhibition in a BRAF^V600E^-driven melanoma model (Long et al., 2019). Thus, MDSCs may play a common role in acquired drug resistance and an important next step is to test whether co-targeting of MDSC recruitment (e.g. through CCR2 inhibition) overrides stroma-driven resistance to atorvastatin treatment. Interestingly, increases in Ly6C^high^ monocytes and monocyte-derived macrophages accompanied by a reduction in alveolar macrophages (AMs) in the TME of advanced KRAS/TP53 lung tumours have been reported recently (Casanova-Acebes et al., 2021), suggesting that a switch from AM-like TAMs to monocyte-derived TAMs is a common feature of TAMs during lung adenocarcinoma progression in mouse models.

Although interactions between MDSCs and Tregs in the TME are well established (Kumar et al., 2016), their functional relationship in our model is unclear. Tumour-infiltrating MDSCs reportedly produce CC chemokines to attract Tregs to the TME (Schlecker et al., 2012), but the distribution of Tregs and M-MDSCs in the atorvastatin-treated KRAS^G12D^ tumours did not overlap (Fig. 5E and Fig. 6D). Rather, the desmoplastic stroma with extracellular matrix-dense architecture seems to restrict the distribution of Tregs in the TA-TLS (Fig. 5D), as previously reported (Salmon et al., 2012), suggesting that Tregs are recruited independently of MDSCs, and that these two immunosuppressive cell types function in distinct compartments. Interestingly, Tregs in the TA-TLS in the atorvastatin-treated KRAS^G12D^ tumours were often associated with Ki67^+^ proliferating non-Treg cells (Fig. 5F), demonstrating that Tregs cannot completely suppress proliferation of neighbouring conventional T cells. As Treg depletion has been reported to cause influx of T cells to tumour parenchyma (Williams et al., 2016), spatial restriction of the distribution of T cells, rather than direct T-cell inhibition, may be more important for Treg-mediated immunosuppression.

By WES, we show that KRAS^G12D^ tumour progression following atorvastatin treatment is not associated with the acquisition of additional driver mutations. Of note, among the genes mutated in statin-treated tumours (Table S2), *Clcc1* and *Kdm1a* are proposed as mutational drivers by the Pan-Cancer Analysis of Whole Genomes Consortium (https://dcc.icgc.org/releases/PCAWG/driver_mutations) (The ICGC/TCGA Pan-Cancer Analysis of Whole Genomes Consortium et al., 2020). However, non-synonymous mutations in *KDM1A* in human lung adenocarcinoma are as rare (0.53% in TCGA PanCancer Atlas) as *CLCC1*, suggesting that *CLCC1* and *KDM1A* are not mutational drivers in this disease. In addition, *Hmcn1* and *Lrp2* were previously reported as mutated in KRAS^G12D^TP53^null^ mouse lung tumours and are frequently mutated in human lung adenocarcinoma (13-16% in TCGA PanCancer Atlas), but are not considered as mutational drivers. As the accumulation of passenger mutations in large genes is mechanistically plausible (Lawrence et al., 2013), the sizes of *HMCN1* and *LRP2* genes encoding >4500 amino acids may explain their high mutation rates.

The relative reduction in exonic SNVs in atorvastatin-treated tumours (Fig. 7A) implies that atorvastatin interferes with the generation of exonic SNVs. Because high mismatch-repair (MMR) activity has been shown to be responsible for the reduced mutation rates in exonic regions in multiple human cancer types (Frigola et al., 2017), atorvastatin may increase MMR activity, leading to the relative reduction of exonic SNVs. Although our mutational signature analysis did not show consistent contribution of defective MMR signatures to somatic SNVs either in control or atorvastatin tumours, contribution of the mutational signature associated with defective DNA double-strand break (DSB) repair was detected only in control tumours (Fig. S7C). Further studies will be needed to clarify the potential linkage between statin treatment and DNA repair mechanisms, including DSB repair and MMR, in the context of cancer-associated mutagenesis.

Overall, our data confirm the efficacy of statins against TAM lineage cells in the early stages of lung adenocarcinoma, but caution against their long-term use. Our data also suggest that clinical development of other TAM-targeting therapeutics may be hampered by similar drug-resistance problems. Thus, a comprehensive understanding of the mechanistic basis of stroma-driven drug resistance and TAM heterogeneity is needed in order to develop better strategies to effectively target TAMs in lung adenocarcinoma. Because neither the BRAF^V600E^ nor the KRAS^G12D^ model in this study is particularly immunogenic, it will also be interesting to investigate the impact of atorvastatin in immunogenic and progressive lung adenocarcinoma models.

## MATERIALS AND METHODS

### Mice

All animal experiments were performed under UK Home Office Licence authority. *Braf^+/LSL−V600E^*, *Kras^+/LSL−G12D^* and *CCAGCreER^TM^* mice were backcrossed onto the C57BL/6J strain and genotyped as reported (Kamata et al., 2017, 2020). Male and female animals >8 weeks of age were used for experimental procedures. Nasal delivery of Ad5-CMV-Cre or Ad5-mSPC-Cre was performed as described (Kamata et al., 2017, 2020). Atorvastatin (10 mg/kg, Generon) was orally administered once daily for the indicated periods. Lung tissues were processed for Haematoxylin and Eosin (H&E) staining and immunohistochemistry (IHC) as described (Kamata et al., 2020).

### Quantitative histology

Whole-slide images of mouse lung sections acquired on a Vectra^®^Polaris™ scanner (PerkinElmer) were imported into QuPath (Bankhead et al., 2017). Tumour areas were defined as hyperplasia, adenoma, pre-invasive or invasive adenocarcinoma as previously described (Nikitin et al., 2004) and outlined manually as annotated regions. Hyperplasia was identified as focal and diffuse lesions involving alveoli and terminal bronchioles and consisting of relatively uniform atypical cuboidal to columnar cells with dense chromatin. Adenomas were defined by well-circumscribed areas, usually less than 5 µm in diameter, consisting of cuboidal to columnar cells lining alveoli and retaining pre-existing alveolar structure. Compared with adenomas, adenocarcinomas were characterised as showing greater nuclear and cytological atypia, increased proliferation, regional variation in growth pattern, more papillary structures and being over 5 µm in diameter. Invasive adenocarcinomas showed invasion of vessels, large airways or pleura, as well as lymphatic and hematogenous metastases. Relative areas of each annotated region were determined for each animal. Proliferation rates were also calculated for each annotated region using immunofluorescence (IF) labelling of Ki67 and the positive cell detection function in QuPath (Bankhead et al., 2017).

### Flow cytometry

Cell surface marker and intracellular SPC expression was analysed by flow cytometry as described (Kamata et al., 2015, 2020). Primary antibodies used were as follows: anti-mouse CD11b (clone M1/70, Tonbo Biosciences; 1:300), anti-Gr1 (clone RB6-8C5, SouthernBiotech; 1:500), anti-mouse CD11c (clone N418, BioLegend; 1:200), anti-F4/80 (clone BM8, BioLegend; 1:100), anti-Ly6C (clone HK1.4, BioLegend; 1:200), anti-mouse CD45 (clone 30-F11, BioLegend; 1:600), anti-mouse CD4 (clone GK1.5, BioLegend; 1:100), anti-mouse CD8a (clone 53-6.7, BioLegend; 1:200), anti-mouse B220 (clone RA3-6B2, BioLegend; 1:200), anti-mouse CD31 (clone MEK13.3, BioLegend; 1:200) and anti-Sca1 (clone D7, Miltenyi Biotech; 1:300) antibodies. Stromal cell types quantified using this panel were previously described (Kamata et al., 2020). For SPC intracellular staining, surface-stained lung cells were fixed/permeabilised using a BD Cytofix/Cytoperm™ kit (BD Biosciences), according to the manufacturer's instructions, and frozen at −20°C for 24 h. Then, the frozen cells were thawed in a 37°C water bath and stained with an anti-SPC antibody (FL-197, Santa Cruz Biotechnology; 1:100) in BD Perm/Wash™ buffer (BD Biosciences) at 37°C for 45 min followed by AlexaFluor^®^488-conjugated anti-rabbit antibody (Thermo Fisher Scientific; 1:2000) staining in BD Perm/Wash™ buffer at room temperature for 20 min.

### IHC and IF staining

IHC/IF staining was performed on paraformaldehyde-fixed, paraffin-embedded (FFPE) mouse lung sections as described (Kamata et al., 2020). Primary antibodies used for IHC/IF were as follows: anti-MAC2 [CL8942AP, Cedarlane; 1:1000 (for AlexaFluor^®^568-conjugated secondary) or 1:5000 (for HRP-conjugated secondary)], anti-Ki67 (clone SP6, Thermo Fisher Scientific; 1:1000), anti-CD3e (A0452, Dako; 1:1000), anti-B220 (clone RA3-6B2, BioLegend; 1:500), anti-pan-cytokeratin (clone AE1/AE3, Abcam; 1:1000), anti-P-AKT (clone D9E, Cell Signaling Technology; 1:1000) and anti-FoxP3 (clone FJK-16s, Thermo Fisher Scientific; 1:400) antibodies. Antibody validation profiles were provided by respective companies upon purchase. Antigen retrieval was performed by 10 min boiling in citrate (10 mM) buffer (pH 6) for MAC2, Ki67, B220, pan-cytokeratin and P-AKT, or in Tris (10 mM)/EDTA (1 mM) buffer (pH 9) for CD3e and FoxP3. Blocking and secondary staining were performed using ImmPRESS™ HRP anti-Rat IgG (mouse-adsorbed) Polymer Detection Kit (Vector Laboratories), SignalStain^®^ Boost IHC Detection reagent (HRP, mouse) (Cell Signaling Technology) or Novolink™ Polymer Detection System (Leica Biosystems) according to the manufacturers’ instructions. IHC was developed using 3,3′-diaminobenzidine (DAB) solution in Novolink™ Polymer Detection System (Leica Biosystems), whereas dual IF staining was performed using OPAL-520/570 tyramide-fluorescent dyes (PerkinElmer) according to the manufacturer's instructions. Whole-slide images of MAC2 IF were acquired using Vectra^®^Polaris™ and analysed with InForm^®^ software package (Akoya Biosciences) to quantify MAC2^+^ and total lung areas. Confocal imaging of IF staining was performed using an Olympus FV1000 confocal laser scanning system with an inverted IX81 motorised microscope equipped with UPlanSApo 60×/1.35 NA objective (Olympus). Images were deconvoluted using Huygens Essential software (Scientific Volume Imaging) and processed using ImageJ software.

### TUNEL staining

TUNEL staining was performed on FFPE lung sections using an ApopTag^®^ Peroxidase *In Situ* Apoptosis Detection Kit (Merck) according to the manufacturer's instructions, in combination with the use of OPAL520 tyramide-fluorescent dye (PerkinElmer) as a peroxidase substrate. TUNEL-stained sections were boiled in citrate (10 mM) buffer (pH 6) for 10 min, followed by MAC2 IF with AlexaFluor^®^568-conjugated anti-rat secondary (Thermo Fisher Scientific) staining.

### Cell culture

Tumour-associated IMCs were freshly isolated from *Braf^+/LSL−V600E^;CreER^+/0^* mice and cultured in serum-free Dulbecco's modified Eagle medium (DMEM) (Invitrogen) as previously described (Kamata et al., 2015, 2020). Atorvastatin (3.3 µM, Generon), lonafarnib (1-5 µM, Tocris Bioscience), GGTI-298 (8 µM, Tocris Bioscience), ML141 (10 µM, Merck) and/or EHT1864 (1-10 µM, Tocris Bioscience) were added to the serum-free IMC culture for 72 h. IMCs cultured for 96 h in serum-free DMEM were treated with the CCR1 inhibitor J113863 (5 µM, Tocris Bioscience) for 1-24 h as indicated. For membrane protein purification and detergent-insoluble protein analysis, primary IMCs were cultured for 48-72 h in DMEM containing 1% foetal bovine serum (Invitrogen) supplemented with atorvastatin (3.3 µM), epoxomicin (0.05 µM, Sigma-Aldrich) and/or MG132 (3.3 µM, Sigma-Aldrich).

### Protein analysis

Protein lysates and conditioned media (CM) samples were prepared as previously described (Kamata et al., 2015, 2020). Membrane protein purification was performed using a ProteoExtract^®^ Native Membrane Protein Extraction Kit (Merck) according to the manufacturer's instructions. Immunoblotting and enzyme-linked immunosorbent assay (ELISA) were performed as previously described (Kamata et al., 2015, 2020). Primary antibodies used for immunoblotting were as follows: anti-CCL6 (ab83134, Abcam; 1:2000), anti-P-AKT (clone D9E, Cell Signaling Technology; 1:2000), anti-pan-AKT (clone C67E7, Cell Signaling Technology; 1:5000), anti-RAC1 (ARC03, Cytoskeleton, Inc.; 1:2000), anti-RHOA (ARH04, Cytoskeleton, Inc.; 1:2000), anti-CDC42 (ACD03, Cytoskeleton, Inc.; 1:2000), anti-pan-RAS (clone EP1125Y, Merck; 1:2000), anti-E-cadherin (clone 36, BD Biosciences; 1:1000), anti-GAPDH (clone GA1R, Thermo Fisher Scientific; 1:4000) and anti-MAC2 (CL8942AP, Cedarlane; 1:2000) antibodies. Antibody validation profiles were provided by respective companies upon purchase.

### WES

Genomic DNA was extracted from tumour and spleen FFPE tissues using a Qiagen GeneRead DNA FFPE Kit according to the manufacturer's instructions. Then, 180-280 bp DNA fragments prepared from 1 µg of the DNA samples were subjected to exome enrichment using a SureSelectXT Mouse All Exon Kit (Agilent Technologies) according to the manufacturer's protocol. The post-capture amplified libraries were clustered on a cBot cluster generation system using TruSeq PE Cluster Kit v4-cBot-HS (Illumina) according to the manufacturer's instructions and sequenced on the Illumina HiSeq2000 sequencing platform. Raw sequencing data were filtered by discarding any read pairs for which >10% of the bases were uncertain, the proportion of low-quality bases were >50% or adaptor contamination was found, in either of the reads. The remaining paired-end clean reads, for which >93% of the bases showed Phred-scaled quality scores >30, were aligned to the mm9 reference genome using BWA-MEM (0.7.8-r455; http://maq.sourceforge.net; Li and Durbin, 2009). The sequencing depth and the coverage in targeted regions were computed on the final BAM files after sorting with Samtools and marking duplicates with Picard (http://sourceforge.net/projects/picard/). We used GATK to detect SNVs/InDels, and somatic SNVs and InDels were called by MuTect (1.1.4; http://www.broadinstitute.org/cancer/cga/mutect; Cibulskis et al., 2013) and Strelka (v1.0.13; Saunders et al., 2012), respectively, using tumour/spleen samples from the same mouse. The calls were annotated with Annovar (22 March 2015; http://www.openbioinformatics.org/annovar/; Wang et al., 2010), and a list of PAMs was created that contained nonsynonymous SNVs and small exonic InDels.

### *In silico* prediction of MHC binding

We extracted all possible neo-peptide sequences of 8-14 mer length containing single amino-acid substitutions corresponding to the non-synonymous SNVs identified by whole-exome sequencing, and their binding to the classical MHC class Ia molecules, H-2Kb (also known as H2-K1) and H-2Db (also known as H2-D1), in the C57BL/6J strain (Vugmeyster et al., 1998) was predicted using NetMHCpan-4.1 (Reynisson et al., 2020). According to the default setting of the software, prediction scores were expressed as %rank with the thresholds for strong and weak binding set as <0.5% and <2%, respectively. The peptide sequence with the highest %rank among the overlapping peptides for each mutation was selected for statistical analyses.

### Theoretical estimation of neo-antigen frequency

The frequency of predicted neo-antigens in total neo-peptides derived from non-synonymous SNVs was compared with the theoretical rate estimated using the silent mutations as reported (Rooney et al., 2015). Briefly, the count of non-synonymous SNVs predicted to generate neo-epitopes (*B*_obs_) was divided by the total count of non-synonymous SNVs (*N*_obs_) to yield ‘observed’ neo-antigen frequency (*B*_obs_/*N*_obs_). The theoretical (‘expected’) frequency *B*_pred_/*N*_pred_ was calculated by dividing the count of synonymous (silent) SNVs involved in MHC class I-binding peptides (*B*_pred_) with the total count of synonymous SNVs (*N*_pred_). The ratio of the observed *B*_obs_/*N*_obs_ against the expected *B*_pred_/*N*_pred_ represents the relative deviation of the neo-epitope rate from expectation. The ratio <1.0 indicates neo-antigen depletion by immunoediting (Rooney et al., 2015; Rosenthal et al., 2019).

### Mutational signature analysis and testing for neutral evolution

A web-based toolkit Mutalisk (http://mutalisk.org) was used to obtain mutation spectra and refitted with COSMIC mutational signatures (v2; https://cancer.sanger.ac.uk/cosmic/signatures) as described (Lee et al., 2018). Somatic SNVs with the depth of coverage ≥10×, supported by at least five variant reads with VAF ≥0.1, were used to determine whether the SNVs are fitted to the neutral evolution model as described (Williams et al., 2016).

### Statistics

Comparison between any two groups was performed by unpaired Student's *t*-test (two-tailed) for numerical data unless otherwise stated, or χ^2^ test followed by post-hoc analyses for categorical data, as described (Shan and Gerstenberger, 2017).

## Supplementary Material

Supplementary information
