# Supplementary material for: Statins mediate anti- and pro-tumourigenic functions by remodelling the tumour microenvironment
Source: Dis Model Mech. 2022 Jan 4;15(2):dmm049148. doi: 10.1242/dmm.049148 (PMC8749029; doi:10.1242/dmm.049148)
Supplement: Supplementary information [file dmm-15-049148-s1.pdf]

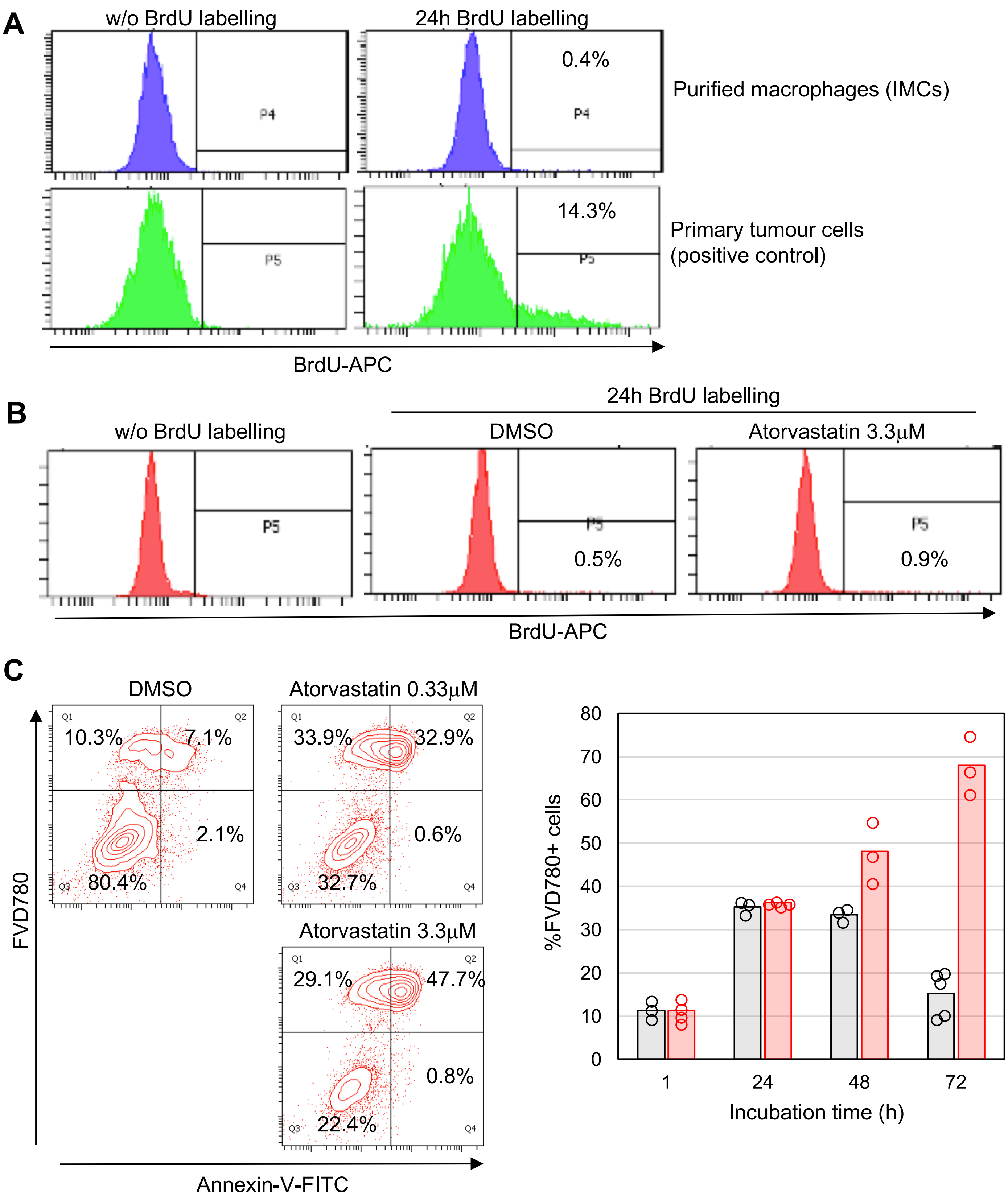

Fig. S1. Ex vivo characterisation of IMCs from BRAF^{V600E} lung tumours. A, Freshly-isolated IMCs (top) and tumour cells (bottom) labelled with BrdU for 24hr were analysed by flow cytometry. **B,** 24h ex vivo culture of purified IMCs with or without atorvastatin (3.3µM in DMEM+2%FCS), followed by 24h BrdU labelling. Incorporated BrdU was analysed as A. **C,** 72h ex vivo culture of purified IMCs with or without atorvastatin (0.33 or 3.3µM in serum-free DMEM), followed by Annexin-V-FITC/fixable viability dye eFluor 780 (FVD780) staining and flow cytometry analysis. Representative flow cytometry plots at 72h culture (left) and time-course of %FVD780-positive IMCs (right, n=3-5) are shown.

Disease Models & Mechanisms • Supplementary information

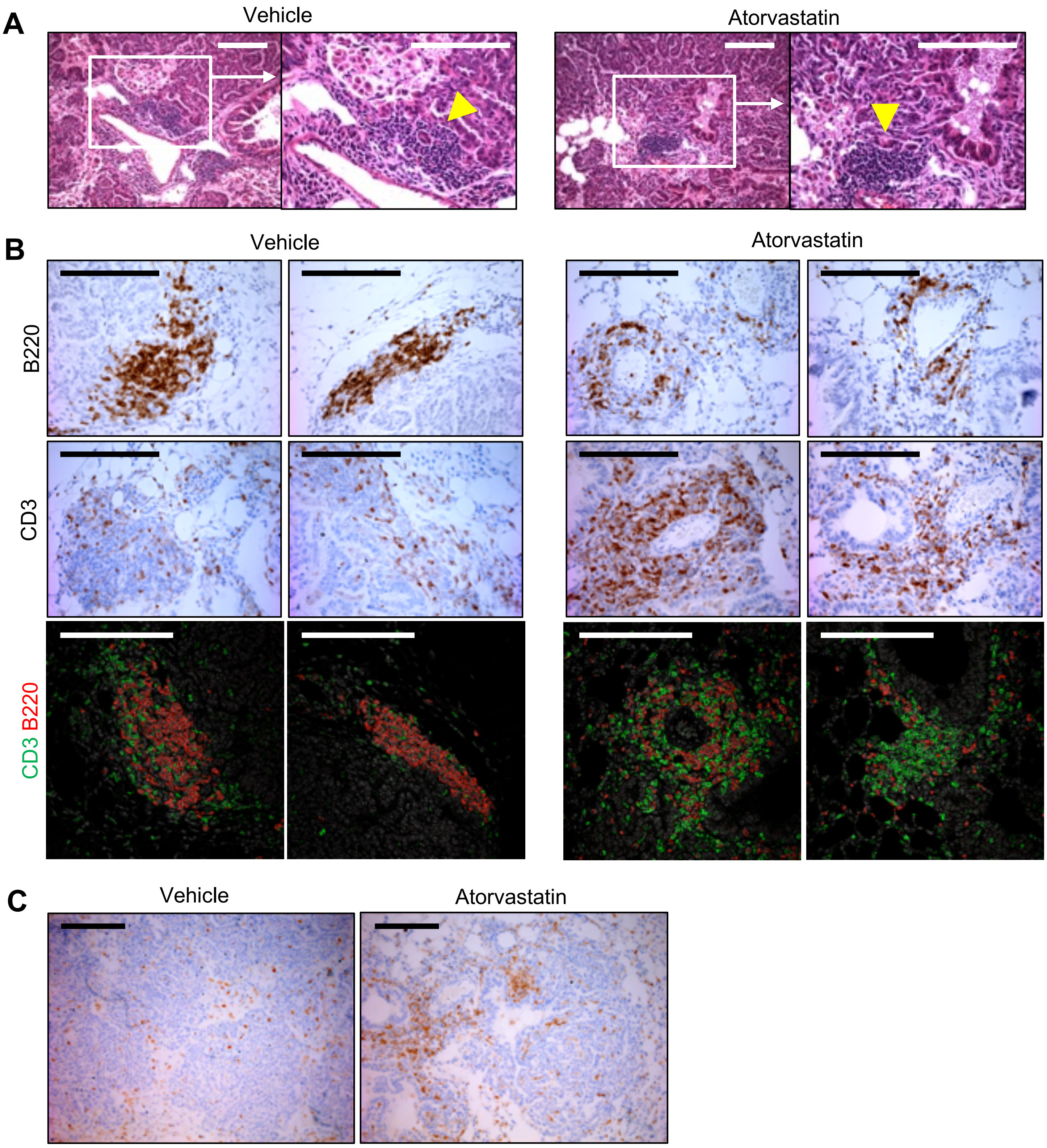

Fig. S2. Histological analysis of TA-TLS in BRAF^{V600E}-driven lung tumours treated with atorvastatin for 5 days. A, H&E staining of vehicle/atorvastatin-treated BRAF^{V600E} tumours. Boxed areas are enlarged in high magnification images. Arrow heads indicate TA-TLS. **B,** B220/CD3 IHC (top and middle) and dual IF (bottom) of TA-TLS dtected in vehicle/atorvastatin-treated BRAF^{V600E} tumours. **C,** CD3 IHC of vehicle/atorvastatin-treated BRAF^{V600E} tumours at a lower magnification, showing intra-tumour T cells. Scale bars = 100μm.

Disease Models & Mechanisms • Supplementary information

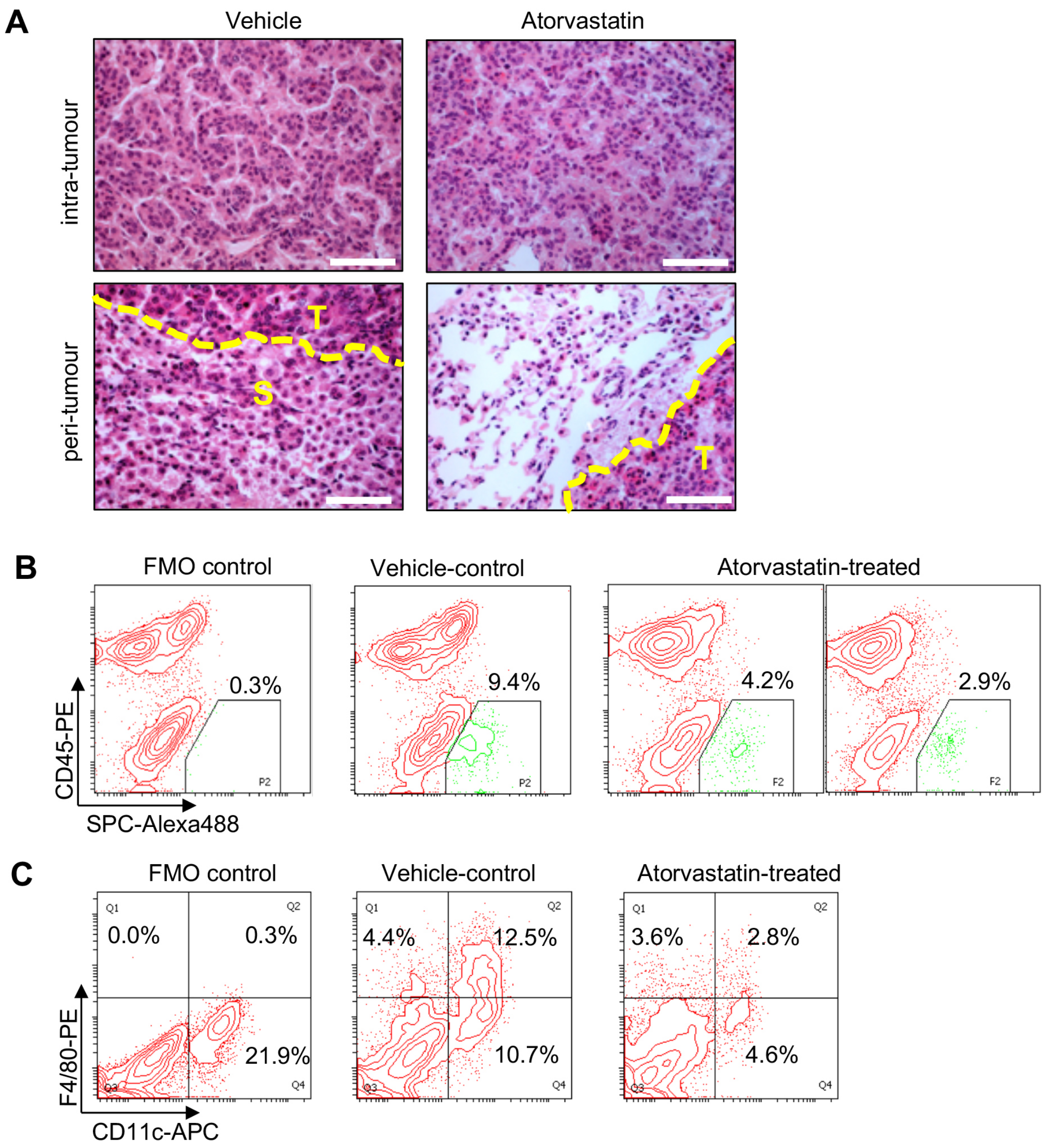

Fig. S3. Histological and flow cytometry analyses of KRASG12D-driven lung tumours treated with atorvastatin for 8 weeks. A, H&E staining of vehicle/atorvastatin-treated KRASG12D tumours. Intra-tumour (top) and peri-tumour (bottom) areas are shown. Tumour (T)–stroma (S) borders are indicated as dotted lines. Scale bars = 50µm. **B and C,** Flow cytometry analysis of vehicle/atorvastatin-treated KRASG12D tumours for CD45-SPC+ cells (B) and CD11c+F4/80+ cells (C). Fluorescence-minus-one (FMO) controls were used to set SPC+and F4/80+ gates.

Disease Models & Mechanisms • Supplementary information

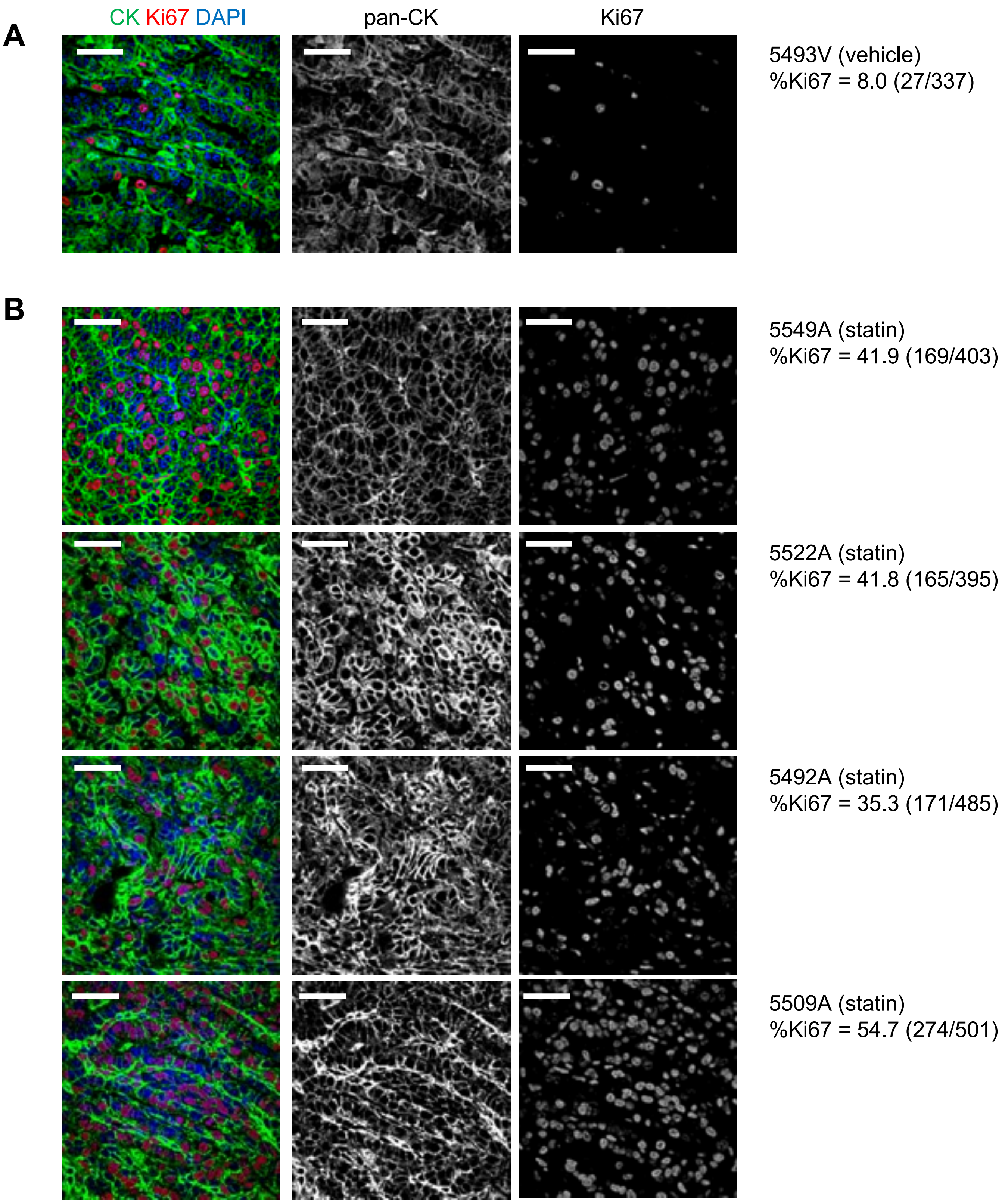

Fig. S4. Ki67 immunofluorescence of KRAS^{G12D}-driven lung tumours developed under long-term atorvastatin treatment. Confocal imaging of Ki67/pan-cytokeratin (AE1/AE3) dual IF staining at end-stages of vehicle/atorvastatin treatment. **A,** Vehicle-control tumour. **B,** Atorvastatin-treated tumours. Scale bars = 50μm.

Disease Models & Mechanisms • Supplementary information

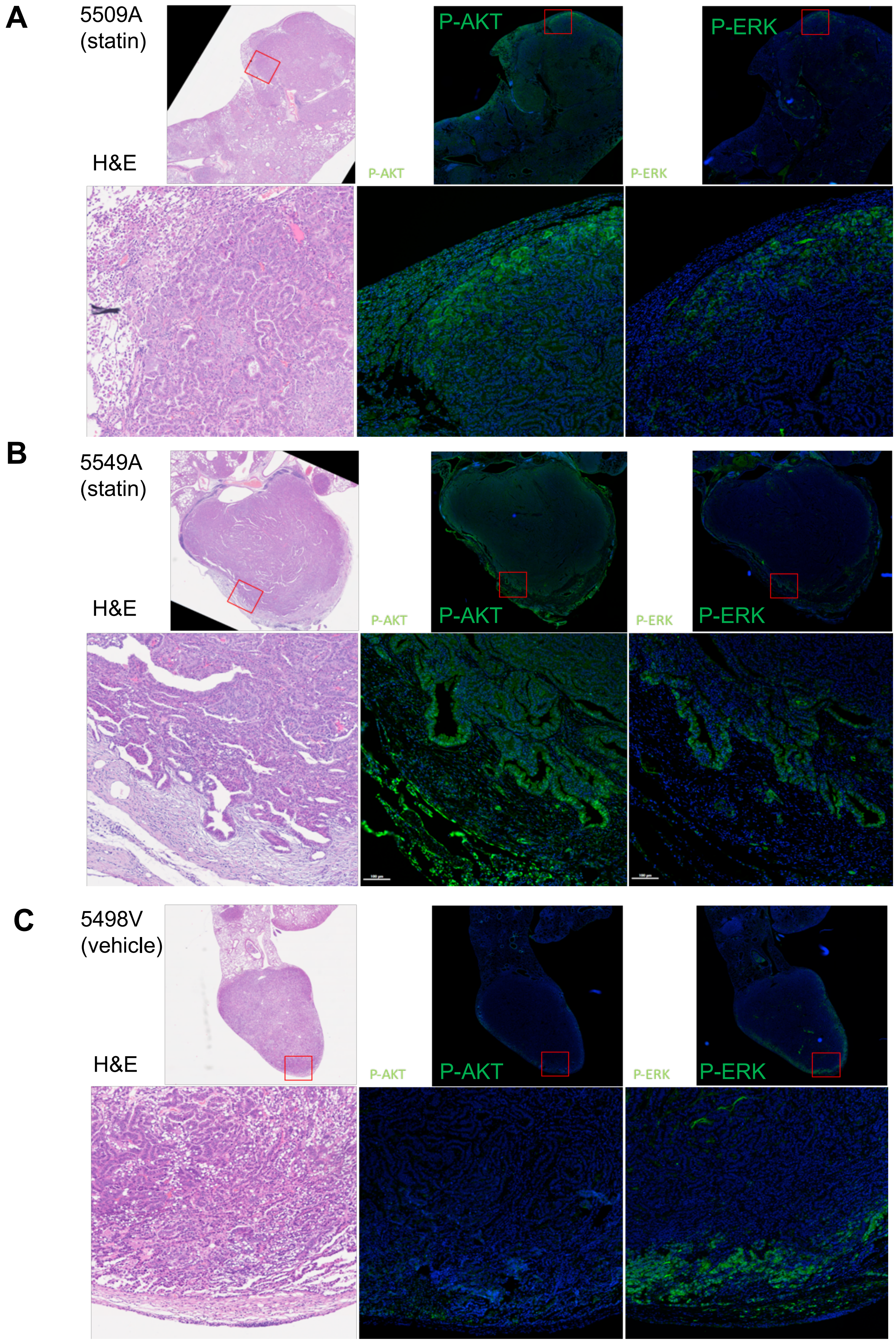

Fig. S5. RAS downstream pathway activation in KRAS^{G12D} tumours following long-term statin treatment. H&E staining (left), P-AKT IF (middle) and P-ERK IF (right) of KRAS^{G12D} lung tumours developed during long-term treatment with atorvastatin (**A**, 5509A and **B**, 5549A) or vehicle (**C**, 5498V). Red boxed areas in the top panels are enlarged in the bottom panels.

Disease Models & Mechanisms • Supplementary information

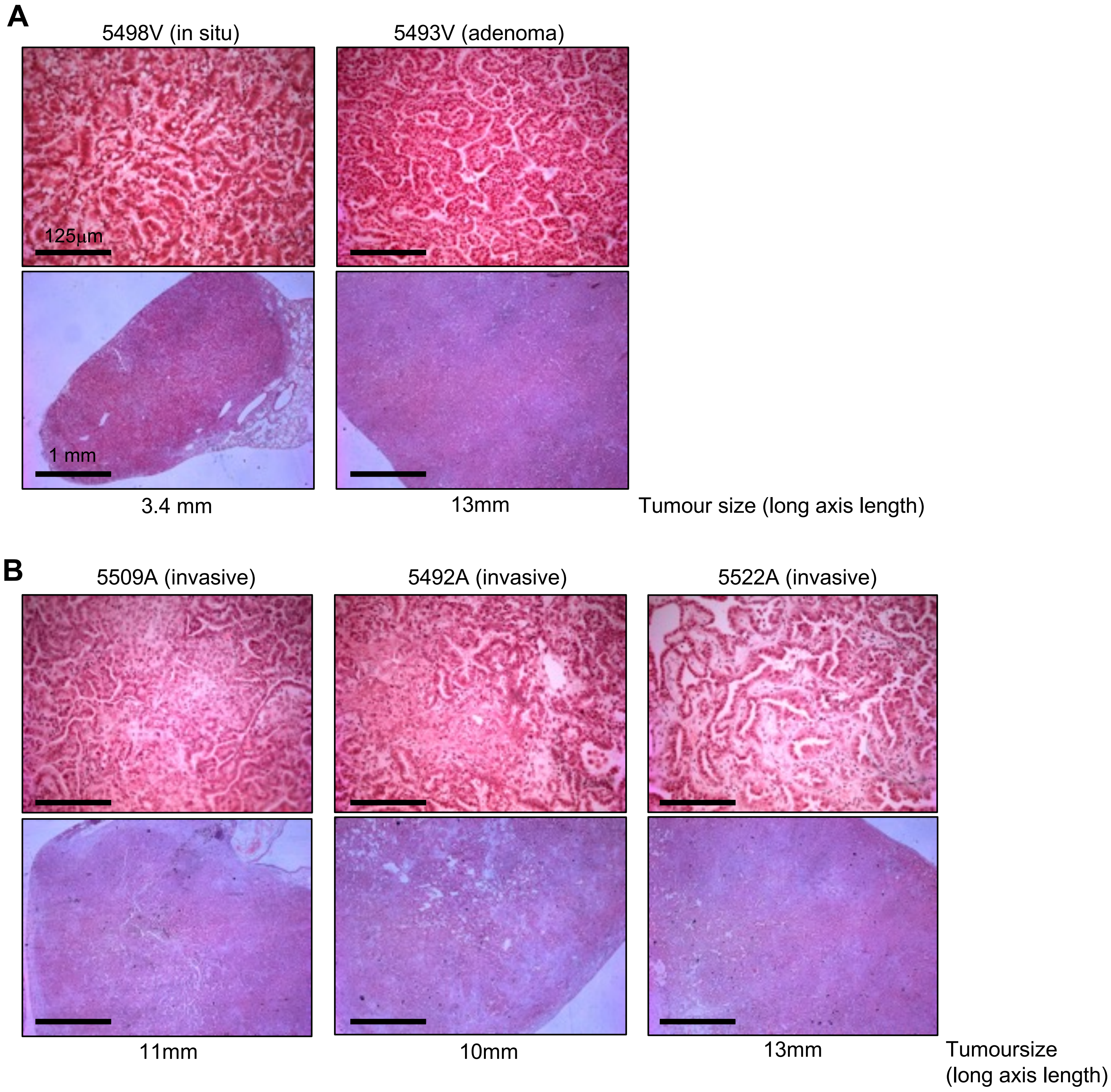

Fig. S6. Histological characteristics of KRAS^{G12D} tumours used for whole-exome sequencing. H&E staining of lung tumours used for genomic DNA extraction for whole-exome sequencing. **A,** Vehicle-control tumours. **B,** Atorvastatin-treated tumours. Scale bars = 125μm (top) or 1mm (bottom).

Disease Models & Mechanisms • Supplementary information

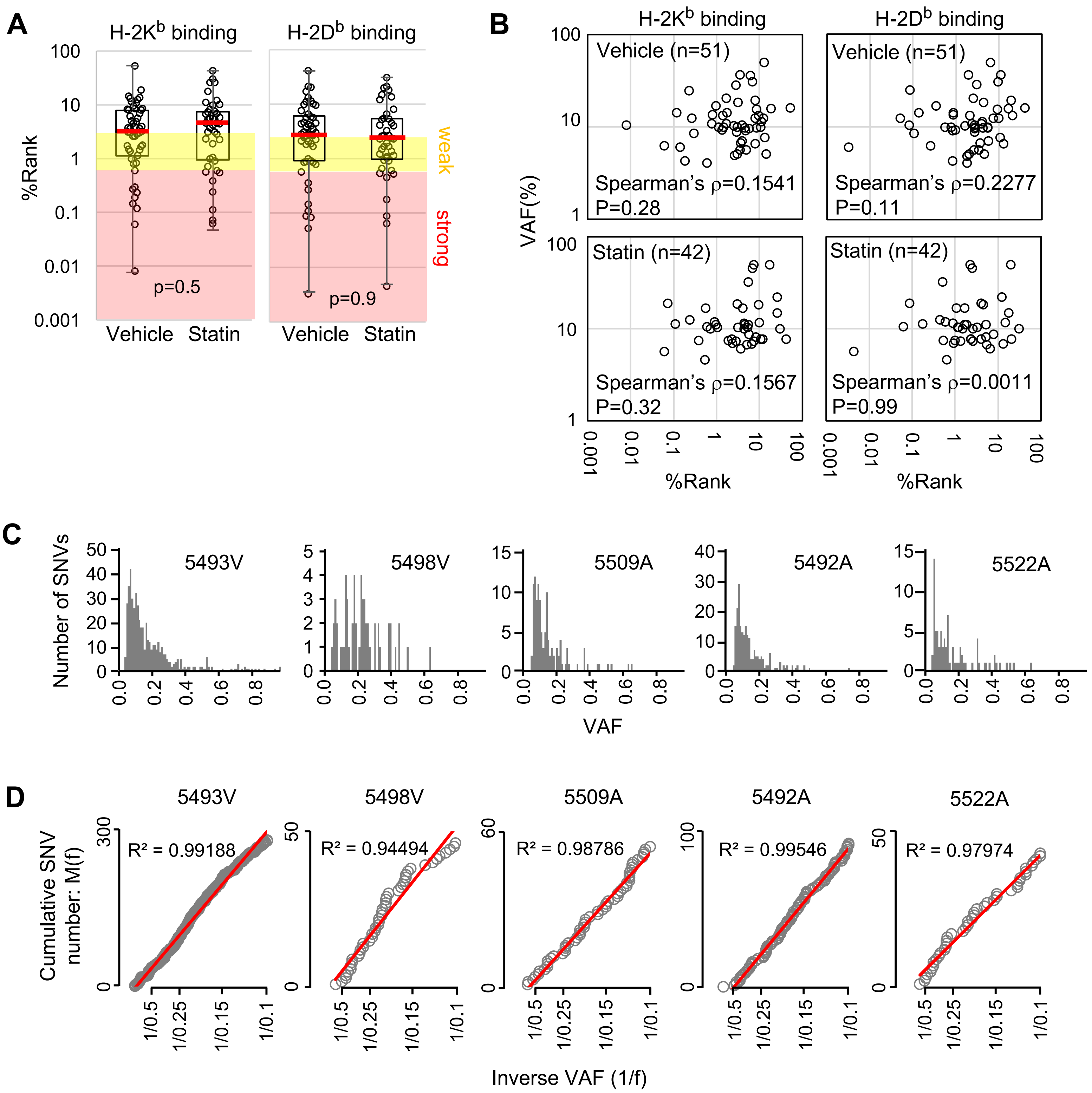

Fig. S7. MHC class I binding prediction and VAF-based estimation of neutral tumour evolution. A, Box plots showing the distribution of %rank of H-2K^b/D^b binding (predicted by NetMHCpan4.1) for the non-synonymous SNV-derived neo-peptides in vehicle/statin-treated tumours. Strong binders <0.5% rank, weak binders <2% rank. **B,** Correlation between variant allele frequencies (VAFs) of non-synonymous SNVs detected in vehicle/statin-treated tumours and predicted H-2K^b/D^b binding (%rank) of the SNV-derived neo-peptides. **C,** The number of somatic SNVs with VAFs ≥ 0.1 in each tumour are indicated as histograms of VAFs. **D,** The data in C are represented as the distribution of M(f), cumulative SNV number with VAF ≥ f. The linear correlations between M(f) and inverse VAF (1/f) in all three atorvastatin-treated tumours predict their fits to the neutral tumour evolution model proposed by Williams et al.

Disease Models & Mechanisms • Supplementary information

Table S1. Non-synonymous mutations indentified in KRASG12D tumours.

Click here to download Table S1

Table S2. Synonymous SNVs in KRASG12D tumours.

Click here to download Table S2

Table S3. MHC class I binding of SNV-derived peptides.

Click here to download Table S3

Disease Models & Mechanisms • Supplementary information
